# Guidance for systematic reviews in journal author instructions: Findings and recommendations for editorial teams

**DOI:** 10.1002/cesm.12050

**Published:** 2024-03-31

**Authors:** Nele S. Pauwels, Muguet Koobasi, Andra Fry, Thomas Vandendriessche, Annie Wittevrongel, Marte Ødegaard

**Affiliations:** ^1^ Knowledge Centre for Health Ghent, Ghent University Ghent University Hospital Ghent Belgium; ^2^ LSE Library London School of Economics and Political Science London UK; ^3^ KU Leuven Libraries, 2Bergen Learning Centre Désiré Collen Leuven Belgium; ^4^ Library of Medicine and Science, University of Oslo Library University of Oslo Oslo Norway

**Keywords:** author guideline, author instruction, editorial recommendation, information specialist, publishing, reporting guideline, systematic review

## Abstract

**Introduction:**

Systematic reviews play a crucial role in informing clinical decision‐making, policy formulation, and evidence‐based practice. However, despite the existence of well‐established guidelines, inadequately executed and reported systematic reviews continue to be published. These highly cited reviews not only pose a threat to the credibility of science but also have substantial implications for medical decision‐making. This study aims to evaluate and recommend improvements to the author instructions of biomedical and health journals concerning the conducting and reporting of systematic reviews.

**Methods:**

A sample of 168 journals was selected based on systematic reviews published between 2020 and 2021, taking into account their Altmetric attention score, citation impact, and mentions in Altmetric Explorer. Author instructions were downloaded, and data extraction was carried out using a standardized web form. Two reviewers independently extracted data, and discrepancies were resolved by a third reviewer. The findings were presented using descriptive statistics, and recommendations for editorial teams were formulated. The protocol is registered with the Open Science Framework Registries (osf. io/bym8d).

**Results:**

One‐third of the journals lack tailored guidance for systematic reviews, as demonstrated by the absence of references to conducting or reporting guidelines, protocol registration, data sharing, and the involvement of an information specialist. Half of the author instructions do not include a dedicated section on systematic reviews, hampering the findability of tailored information. The involvement of information specialists is seldom acknowledged. Ultimately, the absence of an update date in most author instructions raises concerns about the incorporation of the most recent developments and tools for systematic reviews.

**Conclusion:**

Journals that make substantial contributions to synthesizing evidence in biomedicine and health are missing an opportunity to provide clear guidance within their author instructions regarding the conducting and reporting of reliable systematic reviews. This not only fails to inform future authors but also potentially compromises the quality of this frequently published research type. Furthermore, there is a need for greater recognition of the added value of information specialists to the systematic review and publishing processes. This article provides recommendations drawn from the study's observations, aiming to help editorial teams enhance author instructions and, consequently, potentially assisting systematic reviewers in improving the quality of their reviews.

## 1 INTRODUCTION

Systematic reviews play a vital role in evidence‐based medical practice and decision‐making [1]. Given their crucial role in healthcare [1] and the increasing number of published systematic reviews [2], ensuring their quality is of utmost importance. Nonetheless, systematic reviews with poor‐quality search methods are still being published. Such reviews may omit critical information, potentially misguiding healthcare practitioners and decision‐makers [3]. The low quality relates to both the execution [4, 5, 6, 7, 8, 9, 10, 11, 12] and reporting of the search [4, 5, 7, 8]. Besides the wasted resources, this may have serious repercussions for individual patients and our healthcare system as a whole.

Various resources are available to assist researchers in conducting high‐quality systematic reviews. Some organizations have a collaborative editorial system that allows authors to collaborate when publishing a review. Methodological handbooks provide guidance on conducting research effectively [13, 14] while reporting guidelines offer instructions for writing comprehensive systematic review reports [15]. The absence of a clear distinction between conducting and reporting guidelines might lead to their interchangeable use of one for the other purpose. There appears to be a gap between the existence of these guidelines and people's awareness and utilization of them [16]. Journal author instructions could potentially bridge this gap. However, previous assessments by Biocic et al., Goldberg et al., and Rehlicki et al. revealed underperformance in the inclusion of search method requirements in author instructions [17, 18, 19].

Organizations like the International Committee of Medical Journal Editors (ICMJE), the World Association of Medical Editors (WAME), and The Committee on Publication Ethics (COPE) offer recommendations and educational resources for editors and others involved in medical research and publication [20, 21, 22]. However, their recommendations are not mandatory, do not address specific systematic review requirements such as literature search methods and protocol registration, and are often too general about broad subjects such as data sharing, as these organizations typically do not delve extensively into this specific publication type.

In addition to the author instructions provided by journals, information specialists can play a pivotal role in bridging the knowledge gap as they stay updated on current systematic review methods, guidelines, and tools. By engaging with an information specialist at the outset of the review process, researchers can save time and improve efficiency, resulting in a more comprehensive and relevant set of studies included in their review, ultimately leading to more accurate and reliable findings [23, 24, 25, 26, 27, 28].

Overall, although guiding documents, organizations, and information specialists are available to support the quality of systematic reviews, there seems to be a gap in their implementation. The objective of this study is to evaluate the level of systematic reviews guidance provided within the author instructions of biomedicine and health journals, as well as to provide recommendations for editorial organizations and teams.

## 2 METHODS

A cross‐sectional study was conducted on the author instructions of biomedicine and health journals. Its project plan was registered with the Open Science Framework (OSF) Registries (osf. io/bym8d) before data analysis and gives more information about the methods.

### 2.1 Journal subset selection

The subset consists of academic journals that have recently published systematic reviews. To obtain the sample, on August 17, 2022, a search for systematic reviews was conducted on MEDLINE (using PubMed). The search specifically targeted systematic reviews published between 2020 and 2021, covering a span of two full calendar years to accommodate potential publishing fluctuations throughout the year. The search query employed was: “systematic review”[Title] AND 2020/01/01:2021/12/31[Date ‐ Publication] AND “MEDLINE”[Filter]. We exported the systematic reviews from the MEDLINE search and further assessed this set at the journal level. To identify journals with meaningful impact in terms of publishing and outreach, we combined several methods: we selected the top journals based on the number of systematic reviews published yearly, the highest total mentions on Altmetric Explorer (London, United Kingdom), the highest Altmetric attention score, and the highest citation impact on InCites from Clarivate Analytics (London, United Kingdom). On July 10, 2023, the potential predatory status of the journals was assessed using an experimental tool to verify indicators generally associated with predatory publishing [29] and additional manual verifications.

### 2.2 Data collection, extraction, and analysis

On September 23, 2022, the author instructions from the selected journals were downloaded from their respective websites and saved as PDF documents (performed by coauthor MØ). A piloted data extraction form was developed and hosted online. All data categories included in the form can be found in Supporting Information S1: 1. Subsequently, the collected data was transferred to Microsoft Excel (Washington, United States) to reconcile any disparities and to facilitate further analysis. At the start of 2023, discrepancies were resolved by a third reviewer (MK or NSP). The data analysis is presented using descriptive statistics, such as frequency distribution or percentages, and recommendations for editorial teams were formulated based on the study findings.

## 3 RESULTS

A total of 170 unique journals from 23 publishers were included in this study. Two journals were excluded from further analysis: one due to a publisher change between the collection of author instructions and data extraction (*European Journal of Preventive Cardiology*) and the other because the journal exclusively commissions articles (*Sleep Medicine Reviews*). Among the 168 journals, the majority (55 out of 168) were published by Elsevier, followed by 35 Springer journals and 18 Wiley journals. The complete distribution of journals by publisher can be found in Table 1. All data from the author instructions by journal are available in Supporting Information S1: Appendix 1.

**Table 1:** Overview of the journal subset used in this study: number of journals and mentions of publication ethics organizations by publisher.

Publishers' full name	Name used in this paper	Number of journals	COPE	ICMJE	WAME	Other organizations
Elsevier	Elsevier	55	33	53	3	
Springer Nature Group, includes BMC	Springer	35	29	28	5	3 (ENWA)
John Wiley & Sons, Inc.	Wiley	18	12	12	1	3 (ESA, SRCD, Group of Editors of Addiction Journals)
Wolters Kluwer, includes Lippincott Williams & Wilkins	Wolters Kluwer	8	5	7	3	1 (APA)
The BMJ	BMJ	6	5	5	4	
Oxford University Press (OUP), includes Oxford Academic	OUP	6	5	4	1	
Taylor & Francis Group	Taylor & Francis	6	3	5		3 (APA)
Journal of the American Medical Association (JAMA) Network	JAMA	5		5		
Multidisciplinary Digital Publishing Institute (MDPI)	MDPI	5	5	5		
Sage Publications	SAGE	5	4	4		
Public Library of Science (PLOS)	PLOS	4		4		
Frontiers Media SA	Frontiers	3	3	3		
Hindawi Publishing Corporation	Hindawi	2	2	2	1	
AME Publishing Company	AME	1	1	1		
American College of Physicians (ACP Press)	ACP Press	1	1	1		
Cambridge University Press	Cambridge University Press	1		1		
Canadian Medical Association Journal Group (CMAJ)	CMAJ	1	1	1		
International Society of Global Health (ISoGH)	ISoGH	1	1	1		
JMIR Publications	JMIR	1		1		
National Academies Press (NAP)	NAP	1	1			
Verduci Editore	Verduci	1	1	1		1 (CSE)
Via Medica	Via Medica	1	1	1		
Washington DC: American Society of Hematology (ASH)	ASH	1		1		
TOTAL publishers: 23		TOTAL journals: 168	TOTAL COPE mentions: 113	TOTAL ICMJE mentions: 146	TOTAL WAME mentions: 18	TOTAL other mentions: 12

Abbreviations: APA, American Psychological Association; COPE, Committee on Publication Ethics; CSE, Council of Science Editors; ENWA, European Medical Writers Association; ICMJE, International Committee of Medical Journal Editors; SRCD, Society for Research in Child Development; ESA, Ecological Society of America; WAME, World Association of Medical Editors.

### 3.1 Screening for predatory journals

Based on the experimental screening tool developed by Jaques et al. [29] and subsequent manual analysis, four journals required further evaluation. The following three journals were de‐listed from the Web of Science Core Collection by Clarivate, following an announcement in March 2023 [30]: *International Journal of Environmental Research and Public Health* (published by MDPI), *BioMed Research International* (published by Hindawi), and *Annals of Palliative Medicine* (published by AME). The *International Journal of Environmental Research and Public Health* was also excluded from DOAJ in May 2023 due to suspected editorial misconduct by its publisher. However, these journals were not flagged as predatory at the time of sampling. Therefore, they are included in our analysis.

### 3.2 Reference to editorial organizations in the author instructions

Given that editorial organizations provide recommendations and educational resources, we investigated how frequently they were mentioned within the author instructions (Table 1). As shown in Table 1, ICMJE [22] was the most frequently cited organization, appearing in the majority of the surveyed instructions with 146 mentions, followed by COPE [20] with 113 mentions. In addition, some editorial teams indicated that they have developed their own policies for which they assessed compliance. However, among the 168 journals evaluated, nine made no reference to any editorial committee within their author instructions.

The above‐mentioned editorial organizations provide guidance on a wide range of topics, although this was not always explicitly outlined in the author instructions. Specifically, out of the 159 journals referring to an organization, 40 did not specify the role or commitment of any particular organization. However, in 106 of these journals, a reference was made regarding authorship policies. Moreover, out of the 159 journals, 29 referred to these organizations for guidance on methods, 35 for data sharing, and 29 for peer review. References were also made for guidance on conflicts of interest and research integrity, including addressing plagiarism. It is worth noting that references to these organizations were often brief and lacked specificity. For instance, while data sharing was mentioned in a general sense, none specifically addressed it within the context of systematic reviews.

### 3.3 Availability of author instructions tailored for systematic reviews

Out of the 168 journal instructions analyzed, only 63 of them included a separate heading, section, or paragraph specifically dedicated to systematic reviews, despite all these journals publishing this type of review. This indicates that 62% of the journals did not provide specific guidance for systematic reviews. Additionally, 14 journals from nine different publishers incorporated this information into general guidance for original research, simply stating that “systematic reviews are reported as original research.” These findings suggest a potential gap in the support offered by journals to systematic review authors.

### 3.4 Author instructions about conducting and reporting systematic reviews

Among the author instructions we analyzed, we found that only a minority (17 out of 168) explicitly mentioned that authors must adhere to a methodological guideline— these conducting guidelines focus on the design and execution of a systematic review—and indicated that compliance was compulsory (Table 2). In seven other journals, compliance with a methodological guideline was recommended rather than mandated (Table 2), implying its optional nature. The Cochrane Handbook for Systematic Reviews of Interventions was mentioned in the majority of the author instructions (Table 2).

**Table 2:** Journals mentioning conducting guidance for systematic reviews in the author instructions.

Guidance for conducting systematic reviews (reference number)	Compulsory to follow guidance	Optional to follow guidance
Cochrane Handbook for Systematic Reviews of Interventions [13]	7 journals (1 AME, 3 Wiley, 3 Frontiers)	6 journals (2 Springer, 4 Elsevier)
Joanna Briggs Institute (JBI) Manual for Evidence Synthesis [14]	2 journals (1 Wiley, 1 Wolters Kluwer)	‐
Campbell Collaboration [31]	3 journals (Frontiers)	‐
COnsensus‐based Standards for the selection of health Measurement Instruments (COSMIN) [32]	1 journal (Wiley)	‐
Other	7 journals (Elsevier: Lancet's formatting guidelines for systematic reviews and meta‐analysis [33])	1 journal (Wiley: article of Wille‐Jørgensen et al [34])
TOTAL	20 mentions (17 journals)	7 mentions (7 journals)

**FIGURE 1:**
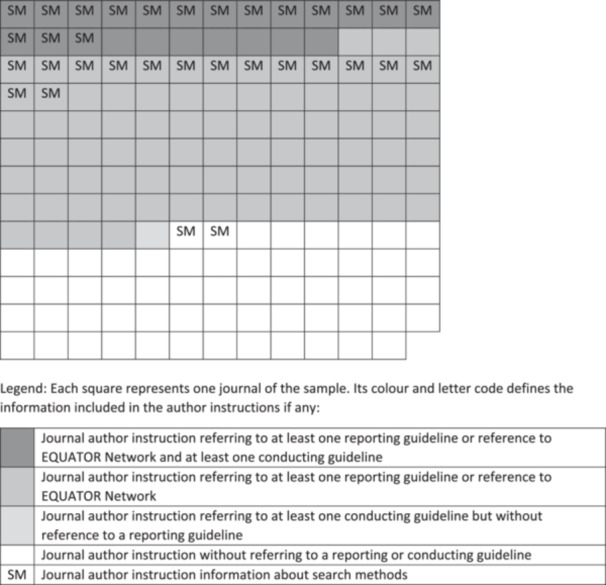
Visualization of mentions of conducting and reporting guidance in the sampled journals.

In 108 out of 168 instructions, at least one reporting guideline—which assists authors in presenting comprehensive details about the methodology employed and the results obtained—was cited, with PRISMA [15] being the most frequently referenced standard (Table 3). Some journals referred to other reporting guidelines (Table 3), but always in addition to PRISMA and/or EQUATOR. A process to validate compliance with the reporting guideline was noted in 42 of the analyzed journals. In the majority of these cases (88%), authors were required to include the reporting checklist during manuscript submission. In the remaining 12%, the checklist was requested as supplementary material. Out of the 168 examined journals, 32 from 10 different publishers provided comprehensive information in the author instructions themselves regarding reporting of search methods, such as the search strategy (Table 4). All except one of these 32 journals (*Trauma Violence & Abuse*) also included at least one reporting standard or a reference to the EQUATOR network in their author instructions. Figure 1 represents a visualization of the mentions of conducting and reporting guidance in our journal subset, illustrating that 34% of the journals (58 out of 168) provided no guidance at all about these aspects.

**Table 3:** Reporting guidance for systematic reviews mentioned in the author instructions.

Guidance for reporting a systematic review	Required to follow the guidance (compulsory)	Recommended to follow the guidance (optional)
Preferred Reporting Items for Systematic Reviews and Met‐Analysis (PRISMA) [15]	73 journals (27 Elsevier, 8 Wiley, 5 JAMA, 5 MDPI, 4 Wolters Kluwer, 3 BMJ, 3 Frontiers, 3 PLOS, 2 OUP, 2 SAGE, 2 Springer, 1 AME, 1 ACP, 1 BMC, 1 Cambridge University Press, 1 CMAJ Group, 1 ISoGH, 1 JMIR, 1 Taylor & Francis, 1 Verduci)	30 journals (17 Springer, 4 Elsevier, 3 BMC journals, 2 Hindawi, 2 Taylor & Francis, 1 OUP, 1 Wiley)
PRISMA for protocols [35]	7 journals (2 Springer, 1 BMJ, 1 Elsevier, 1 Taylor & Francis, 1 Wolters Kluwer, 1 PLOS)	19 journals (16 Springer, 2 BMJ, 1 Wolters Kluwer)
PRISMA for abstracts [15]	3 journals (2 PLOS, 1 Springer)	‐
PRISMA for searching [36]	0	0
PERSiST guidance for implementing PRISMA in Exercise, Rehabilitation, Sport medicine and SporTs science [37]	‐	1 journal (BMJ)
Enhancing the QUAlity and Transparency Of health Research (EQUATOR) [38, 39]	32 journals (14 Elsevier, 5 JAMA, 3 PLOS, 2 Wolters Kluwer, 2 SAGE, 2 Wiley, 1 ACP, 1 BMJ, 1 ISoGH, 1 JMIR)	20 journals (14 Springer Nature, 2 BMC, 2 Wolters Kluwer, 1 Elsevier, 1 OUP)
Meta‐Analysis of Observational Studies in Epidemiology (MOOSE) [40]	17 journals (5 JAMA, 4 Elsevier, 2 Wolters Kluwer, 2 Wiley, 1 ACP, 1 BMJ, 1 Cambridge University Press, 1 OUP)	3 journals (1 OUP, 1 Springer, 1 Wiley)
The Methodological Expectations of Cochrane Intervention Reviews (MECIR) [41]	1 journal (Wiley)	‐
The Institute of Medicine (IOM) [42]	1 journal(Elsevier)	
Campbell Collaboration [31]	3 journal (Frontiers)	‐
Quality of Reporting of Meta‐analyses of randomized controlled trials (QUOROM) [43]	‐	1 journal (Wiley)
Enhancing transparency in reporting the synthesis of qualitative research (ENTREQ) [44]	2 journals (1 ACP, 1 SAGE)	‐
Synthesis Without Meta‐analysis in systematic reviews (SWiM) [45]	1 journal (Wiley)	‐
Realist And Meta‐narrative Evidence Syntheses: Evolving Standards (RAMESES) [46, 47]	1 journal (SAGE)	‐
TOTAL	141 mentions of a reporting guideline as a requirement (77 journals)	74 mentions of a reporting guideline as a recommendation (35 journals)

**Table 4:** Quotes from the author instructions referring to search methods, exported in September 2022.

Journal name	Publisher	Section of the author instructions referring to search methods	Guidance for conducting?	Guidance for reporting?
*Addiction*	Wiley	“It is expected that reviews will be ‘systematic’, which means they will set out very clearly the search strategy (including keywords where appropriate),[…].”	No	Yes
*Annals of Internal Medicine*	ACP	“Abstract Structure: […] Data Sources (must include start and end search dates) […]” “Text […] Subheadings should be: Data Sources and Searches […]”	No	Yes
*Archives of Physical Medicine & Rehabilitation*	Elsevier	“For review articles, systematic or narrative, readers should be informed of the rationale and details behind the literature search strategy.”	No	Yes
*Arthroscopy*	Elsevier	“Literature Search: The search strategy (terms, string) should be described with enough detail that it could be reproduced. Indicate which databases were searched. Two or more databases should be used (the combination of MEDLINE, EMBASE, and Cochrane will capture 97% of all relevant studies in Orthopedic Surgery SR/MA). The search should be performed independently by two or more study authors to ensure no omission of potentially relevant subjects and resolution of disagreement in the setting of possible study inclusion.”	Yes	Yes
*BJOG: An International Journal of Obstetrics and Gynecology*	Wiley	“The Search Strategy (described in detail), and tables with the details of the included and excluded studies should be uploaded as online supplementary information only (not to appear in print).”	Yes	Yes
*BMC Infectious Diseases, BMC Public Health*	Springer	“Authors of systematic reviews should also provide a link to an additional file from the ‘Methods’ section, which reproduces all details of the search strategy. For an example of how a search strategy should be presented, see the Cochrane Reviewers’ Handbook.”	Yes	Yes
*British Journal of Psychiatry*	Cambridge University Press	“Please include a structured abstract […] including data sources […]” “Supplementary Material ‐ […] Details of a search strategy employed in a literature review […]” “Review […] ‐ It is important that the Method section clearly describes the search strategy, study selection criteria, and synthesis approach in sufficient detail to ensure the method can be replicated to extract the same data with the same or similar analysis. This should include information about the protocol registration, review software, data sources (bibliographic databases such as PubMed/MEDLINE, Embase, CINAHL, PsycINFO, and reference lists from journals or books), MeSH and free text search terms and filters, dates included in the search, screening process, language limitations, inclusion and exclusion criteria, study selection and synthesis approach. To ensure a comprehensive review of the literature, we encourage consideration of publications in non‐English languages. Ideally, the search should be as current as possible with the search date noted in the manuscript. […] Supplementary tables, figures, and data should include (in this order): 1. PRISMA‐P (or equivalent) Table 2. Search strings used for various platforms such as MEDLINE, Scopus, etc. 3. PICOS table (if relevant) […]”	No	Yes
*British Journal of Sports Medicine*	BMJ	“Systematic reviews provide Level One evidence; they form a critical part of the literature: […] – The literature search should have been completed within 12 months of manuscript submission.”	No	Yes
*Chest*	Elsevier	“A systematic review involves several steps that can be described in a protocol: […] using several search engines for searches (e.g., PubMed‐MEDLINE, EMBASE, Scopus, Cochrane library) […].”	Yes	Yes
*Clinical Microbiology and Infection*	Elsevier	“Search strategy: Databases searched and search string adapted for each database should be presented (possibly as supplementary material). Study flowchart (in Results) should start transparently from the results of the described search strategy. Restrictions on study years, publication status, or language should be avoided or justified.”	Yes	Yes
*Cochrane Database of Systematic Reviews*	Elsevier	Full section on search methods in the Methodological Expectations of Cochrane Intervention Reviews (MECIR): “1.26 Search methods for identification of studies”	Yes	Yes
*Globalization and Health*	Springer	“Authors of systematic reviews should also provide a link to an additional file from the ‘Methods’ section, which reproduces all details of the search strategy. For an example of how a search strategy should be presented, see the Cochrane Reviewers’ Handbook.”	No	Yes
*Influenza and Other Respiratory Viruses*	Wiley	On reporting: “Literature search and study selection: Guidelines encourage a comprehensive description of the literature search such that it is reproducible.” And “Yet, SRs of surgical interventions frequently require the inclusion of nonrandomized evidence. One of the first problems encountered is the literature search as standard strategies often miss relevant studies because of uncertainty surrounding the use of appropriate search terms, and in turn, results in the retrieval of a large number of irrelevant records. Methodological filters are available to help minimize this, but have yet to be widely implemented.”	Yes	Yes
*JAMA*	JAMA	“The search methods should be described in sufficient detail so the search can be reproduced based on the information provided in the manuscript. A summary of the methods of the literature search including this information should be included in the main article; details can be included in an online‐only supplement.”	No	Yes
*JAMA Internal Medicine, JAMA Pediatrics and JAMA Psychiatry*	JAMA	“Methods/literature search: The literature search should be as current as possible, ideally with end dates within a month or two before manuscript submission. A search of the primary literature should be conducted, including multiple bibliographic databases (eg, PubMed/MEDLINE, Embase, CINAHL, PsycINFO). This can be facilitated by collaborating with a medical librarian to help with the search. Briefly describe characteristics of the literature searched and included in the review, following the PRISMA reporting guidelines, including the bibliographic databases and other sources searched, search terms used, dates included in the search, date the literature search was conducted, screening process, language limitations, and inclusion and exclusion criteria.”	No	Yes
*Journal of the American Academy of Child and Adolescent Psychiatry*	Elsevier	“Review articles should provide a critical assessment of the literature and include the search and selection criteria for data sources.”	No	Yes
*Lancet, Lancet Child & Adolescent Health, Lancet Digital Health, Lancet Gastroenterology & Hepatology, Lancet Global Health, Lancet Microbe, Lancet Psychiatry, Lancet Respiratory Medicine*	Elsevier	“Methods – Search strategy and selection criteria: Describe the data sources assessed. List databases searched and exact date cutoffs. Provide search terms used for at least one database such that the search could be repeated.”	Yes	Yes
*Laryngoscope*	Wiley	“In brief, Systematic Reviews should include the following: […] – Explicit description of the electronic search strategy and databases used (at least 3), including Mesh titles, dates of inclusion, and the names of those performing the search Report of the results of the search, the studies screened, and the studies included […].”	No	Yes
*Mayo Clinic Proceedings*	Elsevier	“Authors are strongly encouraged to describe within the abstract and manuscript text the methods used to focus their search of the literature (eg, PubMed, MEDLINE), the search terms used, and the date limitations of the search.”	No	Yes
*Nutrition Reviews*	OUP	“Methods used to review and evaluate the literature using standardized procedures. This should include the databases used for the review, the key search terms, the criteria for excluding or including previous studies, and how the studies were evaluated and by whom.”	No	Yes
*Palliative Medicine*	SAGE	“All reviews should include sufficient detail on review question, inclusion and exclusion criteria, search strategies, data extraction and synthesis methods (as appropriate to the review design) for the study to be replicated. […] Data sources: State the data sources used (including years searched).”	No	Yes
*Trauma Violence & Abuse*	SAGE	“Each manuscript must: include […] criteria for inclusion, how research studies were identified […]”	No	No

In conclusion, our findings indicate that while only 14% of the analyzed journals explicitly refer to methodological guidelines for conducting a review, a much higher proportion (64%) mention a reporting guideline. This highlights the importance of transparent and comprehensive reporting in scholarly publishing, but conducting and reporting a high‐quality systematic review go hand in hand.

### 3.5 Author instructions about specific aspects of systematic reviews, such as protocol registration, data sharing, and the involvement of an information specialist

**FIGURE 2:**
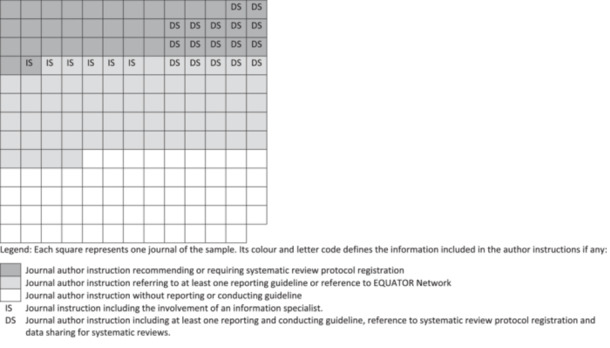
Visualization of the frequency of guidance tailored toward systematic reviews in terms of protocol registration, data sharing, and involvement of an information specialist in the journals' sample.

Out of a total of 168 journals, 41 mentioned protocol registration in relation to a systematic review (Figure 2). Among these, 21 journals recommended the registration of a protocol, leaving the decision up to the authors. Twenty journals stipulated that the registration of a protocol was mandatory. When protocol registration was included in the author instructions, guidance about reporting guidelines was also present (Figure 2). Out of 168 examined journals, 15 of them provided specific recommendations for data sharing concerning the search methods aspect (Figure 2). Interestingly, six out of 15 journals encourage authors to preserve their search strings in SearchRxiv [48], a platform launched in December 2021 for reporting, storing, and sharing search strategies. Of the 168 journals analyzed, only six recommended that authors include the assistance of an information specialist during the search strategy development process (Figure 2).

### 3.6 Update date mentioned in author instructions

In 27 out of 168 author instructions, a publication or an update date was mentioned. Of these 27 instructions, 15 indicated the last update in 2022, the same year as the study. Notably, 141 instructions lack a creation or update date, which leaves authors uncertain about whether the instructions have been revised to reflect the latest developments.

### 3.7 Recommendations for enhancing author instructions derived from our study findings

Table 5 provides a clear outline of six crucial recommendations derived from our study findings, all of which imply mandatory implementation for the systematic reviewer. It also includes supplementary information, which are items that can be readily integrated into the author instructions. These recommendations encompass actionable directives tailored for editorial teams. By adhering to these directives, teams can improve the effectiveness of their author instructions, avoid potential pitfalls, and provide supplementary contextual information.

**Table 5:** Recommendations for enhancing author instructions derived from our study findings.

#	Recommendation for enhancing author instructions	Supplementary information	Action items for editorial teams, including more context and implementation advice
1	*Include a distinct and dedicated section for systematic reviews*.	‐ A section entitled “Systematic Reviews” outlines the essential stages of conducting and reporting a systematic review. These include protocol registration, search methods, study selection, data extraction, risk of bias assessment or critical appraisal, certainty of evidence assessment, evidence synthesis, reporting, and data sharing. ‐ Tailored information for a systematic review, such as conducting and reporting guidelines, protocol registration, data sharing, and the involvement of an information specialist (as in Recommendations 2–6). ‐ A statement addressing publication bias by considering reviews with negative or inconclusive results.	‐ Add more information about the stages of a systematic review, aligning with established international standards, to help authors gain a thorough understanding of each stage before conducting and submitting their systematic reviews. ‐ Avoid using a generic category like “Review”, or a generic statement such as “a systematic review will be considered as original research”. ‐ Address publication bias by considering for publication reviews with negative or inconclusive results.
2	*Clearly differentiate between conducting and reporting stages of systematic reviews*.	‐ A statement, for example: “Conducting guidelines focus on the design and execution of a systematic review while reporting guidelines assist authors in presenting comprehensive details about the methodology employed and the results obtained. Consequently, these two types of guidelines serve distinct purposes and should be treated separately. For instance, a biased systematic review can be thoroughly reported, while an unbiased one may be inadequately reported.”	‐ Incorporate of the differentiation between the conducting and reporting stages.
3	*Provide comprehensive guidance for conducting and reporting systematic reviews*	*Conducting*: ‐ Guidelines that offer a step‐by‐step approach for both beginners and advanced reviewers throughout the entire process of conducting a systematic review. For example, “Cochrane [(13)] and the Joanna Briggs Institute (JBI) [(14)], and more guides are published by Kolaski et al. [(49)].” *Reporting*: ‐ PRISMA statement for systematic reviews, including the PRISMA checklist ‐ PRISMA extension for Protocols ‐ EQUATOR network as a source for specific reporting guidelines based on a review question (e.g.,: PRISMA‐DTA for systematic reviews and meta‐analysis of diagnostic test accuracy studies [(50)]) or type of evidence being covered (e.g.,: ENTREQ for qualitative research reviews [(44)]). ‐ A statement to include the PRISMA checklist (or other reporting checklist, if applicable) as supplementary material for transparency.	‐ Avoid relying solely on one conducting guide and referring to it as the exclusive option considering variations in the policies and support of organizations producing conducting guidelines (some organizations have a collaborative editorial system that allows authors to collaborate when publishing a review). Nonetheless, offering multiple examples of well‐established organizations may assist authors in identifying a guideline that aligns with their individual and institutional needs and goals. ‐ Avoid formulating your own guidance for conducting and reporting systematic reviews and instead refer to established organizations with methodological experts who consistently update their recommendations. ‐ Offer step‐by‐step guidance for both conducting and reporting systematic reviews, and establish a mandatory requirement for reviewers to adhere to this guidance. ‐ Use the PRISMA checklist to verify reporting compliance. ‐ Involve an information specialist in developing the journal's author instructions and during peer review to verify the compliance with reporting guidelines.
4	*Tailor data‐sharing policies and protocol registration examples to systematic reviews*	*Data sharing*: ‐ Specific examples relevant to systematic reviews (e.g., sharing search strategies, template data collection forms, data extracted from included studies, assessments of risk of bias in included studies, and certainty of evidence assessments). ‐ SearchRxiv [(48)], a platform for sharing search strategies, facilitating their reuse. *Protocol registration*: ‐ Reference PROSPERO and Open Science Framework specifically for systematic reviews.	‐ Avoid vague terms like ““data sharing” and clearly tailor examples to systematic reviews. ‐ Differentiate protocol registration for systematic reviews.
5	*Highlight the benefits of involving an information specialist in conducting and reporting systematic reviews*.	‐ The benefits of involving an information specialist in designing and executing effective search strategies, managing references, assisting with data management during screening, and ensuring the comprehensive reporting of these aspects.	‐ Incorporate evidence‐based benefits of involving information specialists [(51)], stressing their role in quality enhancement.
6	*Regularly update journal author instructions with the latest systematic review guidelines, methods, and tools*.	‐ Include the latest update date in the author instructions.	‐ Set a regular updating schedule (e.g., yearly or when new guidelines, methods, or tools emerge). ‐ Involve an information specialist in updating the author instructions.

We strongly advise the inclusion of a dedicated section for systematic reviews within the author instructions. This section should offer comprehensive guidance and illustrative examples to better support and enhance the quality of all stages involved in executing and documenting systematic reviews. Furthermore, it is recommended that editorial teams collaborate with an information specialist. This collaboration is intended to facilitate the creation and maintenance of comprehensive author instructions, ensuring they stay up‐to‐date with evolving best practices.

## 4 DISCUSSION

By increasing the utilization of established guidelines for conducting and reporting systematic reviews, the quality and credibility of published reviews can be improved. Our study examines the guidance provided to authors of systematic reviews in the author instructions of biomedical and health journals and provides recommendations for editorial teams.

Through our journal selection approach, we aimed to curate a collection of influential journals in the field of biomedicine and health. Our study findings suggest that a substantial portion of these journals mention various editorial organizations in their author instructions, signifying their recognition of the importance of adhering to the guidance provided by these organizations. We question whether referencing these editorial organizations is a strategic move to improve the journal's reputation, given the often vague context for which they are cited. It should be noted, however, that the inclusion of such references does not imply membership or certification by the mentioned organizations, and thus those journals could still potentially be associated with predatory practices [52]. Using an experimental tool [29] and manual checks, we evaluated the probability of predatory journals being included in our subset. We did not find evidence of predatory practices at the time of publication for the systematic reviews in our sample. It is important to acknowledge that we cannot verify the common practices of predatory journals from author instructions, such as aggressive article solicitation and the publication of numerous thematic issues without adequate peer review.

In over half of the journals included in our analysis, a dedicated subsection for systematic reviews was absent from the author instructions. Moreover, one‐third of all journals in this sample lacked any guidance related to established conducting or reporting guidelines for systematic reviews, including specific advice on aspects like search methods, protocol registration, and data sharing. Nevertheless, there was a lack of consistency in either recommending or requiring the use of reporting guidelines, and no standardized methods were established to verify adherence to these guidelines. Some organizations and publishers included in this study have released their own advice on conducting systematic reviews since as early as 2019 [53, 54, 55, 56, 57, 58, 59]. These examples might reflect both outdated instructions on one hand and the need for more author guidance on the other. These findings suggest that the information targeted toward systematic reviewers primarily focuses on reporting, lacks cohesion and uniformity, thus highlighting the need for consolidation. Considering these findings, we provided three recommendations (Recommendations 1–3 of the Section 3.7) regarding the provision of a clear and distinct section for systematic reviews in author instructions, including comprehensive guidance on conducting and reporting such reviews.

Without a dedicated section for systematic reviews, there is a risk that the provided information might be too general, making it challenging for systematic reviewers to apply to their own work, especially in aspects like protocol registration and data sharing. A systematic review protocol differs from a clinical study protocol, for example. Surprisingly, only one‐quarter of the examined journals in this study either encouraged or mandated protocol registration for systematic reviews. This is reflected in our recommendation for editorial teams to differentiate protocol registration for systematic reviews and to mention available registries like PROSPERO [60] and Open Science Framework [61]. In clinical research and healthcare, data sharing seems to have gained recognition among relevant stakeholders [62, 63]. However, within the context of a systematic review, “data” encompasses information related to search strategies across all sources, reasons for exclusion during full‐text screening, risk of bias assessment of individual studies, and certainty assessment, among others. Less than 10% of the examined journals provided specific recommendations for data sharing related to search methods in systematic reviews. The need for specification for systematic reviews is reflected in Recommendation 4 of the Section 3.7.

The composition and involvement of team members in shaping and conducting a systematic review can significantly impact its efficiency and outcomes [64]. Only a small fraction of the analyzed journals refers to the role of an information specialist. This specialist can take on various roles within the review process, including serving as a project leader, project manager, literature searcher, reference manager, document supplier, critical appraiser, data extractor, data synthesizer, report writer, disseminator, or primary researcher [51]. This lack of acknowledgment suggests a potential unawareness of the valuable contributions information specialists can make to editorial and systematic review teams. Therefore, we advocate for the involvement of an information specialist at three levels: as a fundamental member of the review project team, as an editorial team member or consultant to assist in drafting and updating journal author instructions, and as a peer reviewer during the publication process (Recommendations 1, 3 and 6 of the Section 3.7).

The absence of an update date for author instructions may indicate that the webpage is considered stable and does not require further updates. However, we raise concerns regarding the potential omission of new and relevant information, such as data sharing of search strategies, and the use of (semi)‐automated or artificial intelligence (AI) tools (for systematic reviews or in general, such as ChatGPT). For example, WAME and COPE published revised recommendations on chatbots and generative AI, which would be valuable and crucial additions to author instructions [65, 66]. The importance of regular updates to ensure coverage of the latest systematic review methods and tools is mentioned in Recommendations 6 (and 3 indirectly) of the Section 3.7.

We noticed two instances in author instructions indicating that only the most clinically impactful systematic reviews and meta‐analyses would be peer‐reviewed and considered for publication. However, this aspect was not part of our main analysis and thus was not quantified separately. These instances are found in *Surgery* and the *Journal of Shoulder and Elbow Surgery*. The consequence of not publishing systematic reviews that are assumed to lack clinical impact, perhaps those with negative findings or conclusions showing gaps in knowledge requiring further research, could lead to publication bias and an incomplete overview and understanding of the available evidence. Such publication policies may be driven by concerns about receiving fewer citations. However, Callaham et al. found that studies with positive results were not necessarily cited more frequently or by more prestigious journals [67]. Publication bias can lead to underestimated values and increased uncertainty in treatment effect estimates [68, 69, 70]. Such bias, both at the clinical trial and the systematic review levels, can result in clinical decisions being based on incomplete or biased information, potentially leading to suboptimal patient outcomes. In contrast, the journal *Systematic Reviews* states its aim to publish the results of all well‐conducted systematic reviews, regardless of their outcome. This observation aligns with Recommendation 1 of the Section 3.7.

These study findings should be considered with the following limitations in mind. First, it is important to acknowledge that online information often consists of interconnected webpages and even published methodological articles. Consequently, extracting complete author instructions posed a challenge for our team, just as it might for authors publishing systematic reviews. Grouping relevant information, such as by publication type, and providing a separate section could enhance accessibility. Second, the author instructions were collected on a specific day (in September 2022) to establish a timestamp for the analysis. However, it is important to recognize that these instructions might have been updated or revised since then. Unfortunately, the absence of publication or update dates—if not dealt with—makes it challenging to assess this aspect for future research. Third, our study does not aim to single out any specific publisher or journal and lacks the power to draw conclusions at the publisher level. Instead, our goal is to raise awareness among editors and editorial teams by making recommendations to enhance author instructions. Fourth, there is an absence of standardizations in terms of methodological guidelines for systematic reviews (compared to reporting guidelines, for example), which results in advice that is more ambiguous and includes numerous examples from established organizations. Fifth, including guidance in the author instructions does not guarantee its implementation, as has been demonstrated previously [16]. However, emergencies, urgent situations, or rapid responses like those during the COVID‐19 pandemic do not justify shortcuts in conducting and reporting systematic reviews [71, 72]. Nevertheless, we are currently observing a significant lack of guidance in author instructions, forcing systematic reviewers to rely on alternative sources of information. Finally, our selection of journals for the study primarily focused on the biomedical and health field due to the sampling source. Consequently, we cannot draw conclusions about other disciplines or more specific areas within medical and health research.

## 5 CONCLUSION

The author instructions provided by journals that make substantial contributions to summarizing evidence in biomedicine and health generally lack tailored and detailed information for conducting and reporting systematic reviews. Furthermore, they generally do not mention information specialists who could offer valuable assistance. This lack of guidance not only fails to inform future authors but also potentially compromises the quality of systematic reviews. Our recommendations aim to bridge this information gap by enhancing and enriching author instructions for systematic reviews in biomedical and health journals.

## AUTHOR CONTRIBUTIONS

**Nele. S Pauwels, Muguet Koobasi, and Marte Ødegaard**: Conceptualization; data curation; formal analysis; investigation; methodology; project administration; supervision; validation; visualization; writing—review & editing. **Nele. S Pauwels and Marte Ødegaard**: Methodology; writing—original draft; writing—review & editing. **Andra Fry, Thomas Vandendriessche, and Annie Wittevrongel**: Formal analysis; methodology; data extraction; writing—review & editing.

## CONFLICT OF INTEREST STATEMENT

The authors declare no conflicts of interest.

## ETHICS STATEMENT

The survey did not involve human or animal research and was not based on sensitive information or personal data of any kind. Therefore, approval from an ethics committee was not required.

## Supporting information

Supporting information.

## Data Availability

The data that supports the findings of this study are available in the supplementary material of this article.

## References

[1] Gopalakrishnan S , Ganeshkumar P . Systematic reviews and meta‐analysis: understanding the best evidence in primary healthcare. J Family Med Prim Care. 2013;2(1):9‐14. 10.4103/2249-4863.109934 24479036 PMC3894019

[2] Ioannidis JPA . The mass production of redundant, misleading, and conflicted systematic reviews and meta‐analyses. Milbank Q. 2016;94(3):485‐514. 10.1111/1468-0009.12210 27620683 PMC5020151

[3] Uttley L , Quintana DS , Montgomery P , et al. The problems with systematic reviews: a living systematic review. J Clin Epidemiol. 2023;156:30‐41. 10.1016/j.jclinepi.2023.01.011 36796736

[4] Faggion Jr. CM , Atieh MA , Park S . Search strategies in systematic reviews in periodontology and implant dentistry. J Clin Periodontol. 2013;40(9):883‐888. 10.1111/jcpe.12132 23834263

[5] Faggion Jr. CM , Huivin R , Aranda L , Pandis N , Alarcon M . The search and selection for primary studies in systematic reviews published in dental journals indexed in MEDLINE was not fully reproducible. J Clin Epidemiol. 2018;98:53‐61. 10.1016/j.jclinepi.2018.02.011 29476922

[6] Franco JVA , Garrote VL , Escobar Liquitay CM , Vietto V . Identification of problems in search strategies in Cochrane Reviews. Res Synth Methods. 2018;9(3):408‐416. 10.1002/jrsm.1302 29761662

[7] Koffel JB , Rethlefsen ML . Reproducibility of search strategies is poor in systematic reviews published in high‐impact pediatrics, cardiology and surgery journals: a cross‐sectional study. PLoS One. 2016;11(9):e0163309. 10.1371/journal.pone.0163309 27669416 PMC5036875

[8] Mullins MM , DeLuca JB , Crepaz N , Lyles CM . Reporting quality of search methods in systematic reviews of HIV behavioral interventions (2000–2010): are the searches clearly explained, systematic and reproducible?: Reporting quality of search methods. Res Synth Methods. 2014;5(2):116‐130. 10.1002/jrsm.1098 26052651 PMC5861495

[9] Opheim E , Andersen PN , Jakobsen M , Aasen B , Kvaal K . Poor quality in systematic reviews on PTSD and EMDR—an examination of search methodology and reporting. Front Psychol. 2019;10:1558. 10.3389/fpsyg.2019.01558 31354575 PMC6630178

[10] Salvador‐Oliván JA , Marco‐Cuenca G , Arquero‐Avilés R . Errors in search strategies used in systematic reviews and their effects on information retrieval. J Med Libr Assoc. 2019;107(2):210‐221. 10.5195/jmla.2019.567 31019390 PMC6466507

[11] Sampson M , McGowan J . Errors in search strategies were identified by type and frequency. J Clin Epidemiol. 2006;59(10):1057.e1‐1057.e9. 10.1016/j.jclinepi.2006.01.007 16980145

[12] Yoshii A , Plaut DA , McGraw KA , Anderson MJ , Wellik KE . Analysis of the reporting of search strategies in Cochrane systematic reviews. J Med Library Assoc: JMLA. 2009;97(1):21‐29. 10.3163/1536-5050.97.1.004 PMC2605027 19158999

[13] Higgins JP , Thomas J , Chandler J , et al. Cochrane Handbook for Systematic Reviews of Interventions. John Wiley & Sons; 2019. 10.1002/14651858.ED000142 PMC10284251 31643080

[14] Aromataris E , Munn Z . JBI Manual for Evidence Synthesis. JBI; 2020.

[15] Page MJ , McKenzie JE , Bossuyt PM , et al. The PRISMA 2020 statement: an updated guideline for reporting systematic reviews. BMJ. 2021;372:n71. 10.1136/bmj.n71 33782057 PMC8005924

[16] Page MJ , Moher D . Evaluations of the uptake and impact of the Preferred Reporting IItems for Systematic reviews and Meta‐Analyses (PRISMA) Statement and extensions: a scoping review. Syst Rev. 2017;6(1):263. 10.1186/s13643-017-0663-8 29258593 PMC5738221

[17] Biocic M , Fidahic M , Puljak L . Reproducibility of search strategies of non‐Cochrane systematic reviews published in anaesthesiology journals is suboptimal: primary methodological study. Br J Anaesth. 2019;122(6):e79‐e81. 10.1016/j.bja.2019.02.014 30916036

[18] Goldberg J , Boyce LM , Soudant C , Godwin K . Assessing journal author guidelines for systematic reviews and meta‐analyses: findings from an institutional sample. J Med Libr Assoc. 2022;110(1):63‐71. 10.5195/jmla.2022.1273 35210964 PMC8830390

[19] Rehlicki D , Plenkovic M , Delac L , Pieper D , Marušić A , Puljak L . Author instructions in biomedical journals infrequently address systematic review reporting and methodology: a cross‐sectional study. J Clin Epidemiol. 2024;166:111218. 10.1016/j.jclinepi.2023.11.008 37993073

[20] Committee on Publication Ethics (COPE). Accessed August 1, 2023. publicationethics.org

[21] World Association of Medical Editors (WAME). Accessed August 1, 2023. wame.org

[22] International Committee of Medical Journal Editors (ICMJE). Accessed August 1, 2023. icmje.org 10.12771/emj.2024.e48 PMC12093628 40704003

[23] Aamodt M , Huurdeman H , Strømme H . Librarian co‐authored systematic reviews are associated with lower risk of bias compared to systematic reviews with acknowledgement of librarians or no participation by librarians. Evid Based Libr and Inf Pract. 2019;14(4):103‐127. 10.18438/eblip29601

[24] Li L , Tian J , Tian H , et al. Network meta‐analyses could be improved by searching more sources and by involving a librarian. J Clin Epidemiol. 2014;67(9):1001‐1007. 10.1016/j.jclinepi.2014.04.003 24841794

[25] Meert D , Torabi N , Costella J . Impact of librarians on reporting of the literature searching component of pediatric systematic reviews. J Med Libr Assoc: JMLA. 2016;104(4):267‐277. 10.3163/1536-5050.104.4.004 27822147 PMC5079487

[26] Rethlefsen ML , Farrell AM , Osterhaus Trzasko LC , Brigham TJ . Librarian co‐authors correlated with higher quality reported search strategies in general internal medicine systematic reviews. J Clin Epidemiol. 2015;68(6):617‐626. 10.1016/j.jclinepi.2014.11.025 25766056

[27] Rethlefsen ML , Murad MH , Livingston EH . Engaging medical librarians to improve the quality of review articles. JAMA. 2014;312(10):999‐1000. 10.1001/jama.2014.9263 25203078

[28] Schellinger J , Sewell K , Bloss JE , Ebron T , Forbes C . The effect of librarian involvement on the quality of systematic reviews in dental medicine. PLoS One. 2021;16(9):e0256833. 10.1371/journal.pone.0256833 34469487 PMC8409615

[29] Jaques C , Trombert A , Zbinden J , Elmers J . An emerging concern in the systematic review process: identifying articles published in predatory journals. presented at: European Association for Health Information and Libraries (EAHIL) conference European Association for Health Information and Libraries (EAHIL) conference Rotterdam. 2022. bium.ch/wp-content/uploads/2022/06/An_emerging_concern_in_systematic_reviews_process-eahil2022.pdf

[30] Web of Science. De‐listed 82 journals, including 15 from Hindawi. March 23, 2023. Accessed August 1, 2023. predatoryreports.org/news/f/web-of-science-de-listed-82-journal-including-15-from-hindawi

[31] Campbell Collaboration. Accessed August 1, 2023. campbellcollaboration.org

[32] Mokkink LB , Prinsen CAC , Bouter LM , Vet HCW , Terwee CB . The COnsensus‐based Standards for the selection of health Measurement INstruments (COSMIN) and how to select an outcome measurement instrument. Braz J Phys Ther. 2016;20:105‐113. 26786084 10.1590/bjpt-rbf.2014.0143 PMC4900032

[33] Systematic reviews and meta‐analyses in The Lancet: formatting guidelines. Accessed August 1, 2023. thelancet.com/pb/assets/raw/Lancet/authors/metaguidelines-1668613510077.pdf

[34] Wille‐Jørgensen P , Renehan AG . Systematic reviews and meta‐analyses in coloproctology: interpretation and potential pitfalls. Colorectal Dis. 2008;10(1):21‐32. 10.1111/j.1463-1318.2007.01421.x 18005187

[35] Moher D , Shamseer L , Clarke M , et al. Preferred reporting items for systematic review and meta‐analysis protocols (PRISMA‐P) 2015 statement. Syst Rev. 2015;4(1):1. 10.1186/2046-4053-4-1 25554246 PMC4320440

[36] Rethlefsen ML , Kirtley S , Waffenschmidt S , et al. PRISMA‐S: an extension to the PRISMA statement for reporting literature searches in systematic reviews. Syst Rev. 2021;10(1):39. 10.1186/s13643-020-01542-z 33499930 PMC7839230

[37] Ardern CL , Büttner F , Andrade R , et al. Implementing the 27 PRISMA 2020 statement items for systematic reviews in the sport and exercise medicine, musculoskeletal rehabilitation and sports science fields: the PERSiST (implementing Prisma in Exercise, Rehabilitation, Sport medicine and SporTs science) guidance. Br J Sports Med. 2022;56(4):175‐195. 10.1136/bjsports-2021-103987 34625401 PMC8862073

[38] Equator Network. Accessed August 1, 2023. equator-network.org

[39] Simera I , Moher D , Hoey J , Schulz KF , Altman DG . The EQUATOR network and reporting guidelines: helping to achieve high standards in reporting health research studies. Maturitas. 2009;63(1):4‐6. 10.1016/j.maturitas.2009.03.011 19372017

[40] Stroup DF . Meta‐analysis of observational studies in epidemiology: a proposal for reporting. Meta‐analysis Of Observational Studies in Epidemiology (MOOSE) group. JAMA. 2000;283(15):2008‐2012. 10.1001/jama.283.15.2008 10789670

[41] Higgins JPT , Lasserson T , Chandler J , Tovey D , Churchill R . *Methodological Expectations of Cochrane Intervention Reviews*. Cochrane; 2016.

[42] Institute of Medicine . Finding What Works in Health Care: Standards for Systematic Reviews. The National Academies Press; 2011:340. 24983062

[43] Moher D , Cook DJ , Eastwood S , Olkin I , Rennie D , Stroup DF . Improving the quality of reports of meta‐analyses of randomised controlled trials: the QUOROM statement. Oncol Res Treat. 2000;23(6):597‐602. 10.1159/000055014 11441269

[44] Tong A , Flemming K , McInnes E , Oliver S , Craig J . Enhancing transparency in reporting the synthesis of qualitative research: ENTREQ. BMC Med Res Methodol. 2012;12(1):181. 10.1186/1471-2288-12-181 23185978 PMC3552766

[45] Campbell M , McKenzie JE , Sowden A , et al. Synthesis without meta‐analysis (SWiM) in systematic reviews: reporting guideline. BMJ. 2020;368:l6890. 10.1136/bmj.l6890 31948937 PMC7190266

[46] Wong G , Greenhalgh T , Westhorp G , Buckingham J , Pawson R . RAMESES publication standards: realist syntheses. BMC Med. 2013;11:1‐14. 23360677 10.1186/1741-7015-11-21 PMC3558331

[47] Wong G , Greenhalgh T , Westhorp G , Buckingham J , Pawson R . RAMESES publication standards: meta‐narrative reviews. J Adv Nurs. 2013;69(5):987‐1004. 23356699 10.1111/jan.12092

[48] SearchRxiv. Accessed August 1, 2023. cabidigitallibrary.org/journal/searchrxiv

[49] Kolaski K , Logan LR , Ioannidis JPA . Guidance to best tools and practices for systematic reviews. Syst Rev. 2023;12(1):96. 10.1186/s13643-023-02255-9 37291658 PMC10248995

[50] McInnes MDF , Moher D , Thombs BD , et al. Preferred reporting items for a systematic review and meta‐analysis of diagnostic test accuracy studies: the PRISMA‐DTA statement. JAMA. 2018;319(4):388‐396. 10.1001/jama.2017.19163 29362800

[51] Beverley CA , Booth A , Bath PA . The role of the information specialist in the systematic review process: a health information case study. Health Inf Libr J. 2003;20(2):65‐74. 10.1046/j.1471-1842.2003.00411.x 12786905

[52] Journals stating that they follow the ICMJE Recommendations. Accessed August 1, 2023. icmje.org/journals-following-the-icmje-recommendations/

[53] Xiao Y , Watson M . Guidance on conducting a systematic literature review. J Plan Educ Res. 2019;39(1):93‐112. 10.1177/0739456x17723971

[54] Carrera‐Rivera A , Ochoa W , Larrinaga F , Lasa G . How‐to conduct a systematic literature review: a quick guide for computer science research. MethodsX. 2022;9:101895. 10.1016/j.mex.2022.101895 36405369 PMC9672331

[55] Khan KS , Kunz R , Kleijnen J , Antes G . Five steps to conducting a systematic review. J R Soc Med. 2003;96(3):118‐121. 10.1177/014107680309600304 12612111 PMC539417

[56] Tawfik GM , Dila KAS , Mohamed MYF , et al. A step by step guide for conducting a systematic review and meta‐analysis with simulation data. Trop Med Health. 2019;47(1):46. 10.1186/s41182-019-0165-6 31388330 PMC6670166

[57] Caldwell PH , Bennett T . Easy guide to conducting a systematic review. J Paediatr Child Health. 2020;56(6):853‐856. 10.1111/jpc.14853 32364273

[58] MacMillan F , McBride KA , George ES , Steiner GZ . Conducting a systematic review: a practical guide. Handbook of Research Methods in Health Social Sciences. Springer Singapore; 2019:805‐826

[59] Zaccagnini M , Li J . How to conduct a systematic review and meta‐analysis: a guide for clinicians. Respir Care. 2023;68:1295‐1308. 10.4187/respcare.10971 37072163 PMC10468159

[60] PROSPERO. Accessed August 1, 2023. crd.york.ac.uk/prospero/

[61] Open Science Framework (OSF). Accessed August 1, 2023. osf.io

[62] Drazen JM , Morrissey S , Malina D , Hamel MB , Campion EW . The importance—and the complexities—of data sharing. N Engl J Med. 2016;375(12):1182‐1183. 10.1056/NEJMe1611027 27653569

[63] Ross JS , Lehman R , Gross CP . The importance of clinical trial data sharing: toward more open science. Circ: Cardiovasc Qual Outcomes. 2012;5(2):238‐240. 10.1161/CIRCOUTCOMES.112.965798 22438465 PMC3318983

[64] Uttley L , Montgomery P . The influence of the team in conducting a systematic review. Syst Rev. 2017;6(1):149. 10.1186/s13643-017-0548-x 28764779 PMC5540536

[65] Lapeña JF . The updated World Association of Medical Editors (WAME) recommendations on chatbots and generative AI in relation to scholarly publications and International Committee of Medical Journal Editors (ICMJE) recommendations for the conduct, reporting, editing, and publication of scholarly work in medical journals. Philipp J Otolaryngol HNS. 2023;38(1):4. 10.32412/pjohns.v38i1.2127

[66] COPE position statement on AI tools. February 13, 2023. Accessed August 1, 2023. publicationethics.org/cope-position-statements/ai-author

[67] Callaham M . Journal prestige, publication bias, and other characteristics associated with citation of published studies in peer‐reviewed journals. JAMA. 2002;287(21):2847‐2850. 10.1001/jama.287.21.2847 12038930

[68] Copas JB , Shi JQ . A sensitivity analysis for publication bias in systematic reviews. Stat Methods Med Res. 2001;10(4):251‐265. 10.1177/096228020101000402 11491412

[69] Kicinski M , Springate DA , Kontopantelis E . Publication bias in meta‐analyses from the Cochrane Database of Systematic Reviews. Stat Med. 2015;34(20):2781‐2793. 10.1002/sim.6525 25988604

[70] Hopewell S , Loudon K , Clarke MJ , Oxman AD , Dickersin K . Publication bias in clinical trials due to statistical significance or direction of trial results. Cochrane Database Syst Rev. 2009;2010:MR000006. 10.1002/14651858.MR000006.pub3 PMC8276556 19160345

[71] Schünemann HJ , Santesso N , Vist GE , et al. Using GRADE in situations of emergencies and urgencies: certainty in evidence and recommendations matters during the COVID‐19 pandemic, now more than ever and no matter what. J Clin Epidemiol. 2020;127:202‐207. 10.1016/j.jclinepi.2020.05.030 32512187 PMC7274969

[72] McDonald S , Turner SL , Nguyen P‐Y , Page MJ , Turner T . Are COVID‐19 systematic reviews up to date and can we tell? A cross‐sectional study. Syst Rev. 2023;12(1):85. 10.1186/s13643-023-02253-x 37202770 PMC10193307

