# Supplementary material for: Guidance for systematic reviews in journal author instructions: Findings and recommendations for editorial teams
Source: Cochrane Evid Synth Methods. 2024 Mar 31;2(4):e12050. doi: 10.1002/cesm.12050 (PMC11795970; doi:10.1002/cesm.12050)
Supplement: Supplementary file 1 — Supporting information. [file CESM-2-e12050-s001.PDF]

Guidance for Systematic Reviews in Journal Author Instructions: Findings and Recommendations for Editorial Teams

Nele S. Pauwels[1], Muguet Koobasi[1], Andra Fry[2], Thomas Vandendriessche[3], Annie Wittevrongel[1] and Marte Ødegaard[4]

[1] Knowledge Centre for Health Ghent, Ghent University, Ghent University Hospital, Ghent, Belgium

[2] LSE Library, London School of Economics and Political Science, London, UK

[3] KU Leuven Libraries, 2Bergen, Learning Centre Désiré Collen, Leuven, Belgium

[4] Library of Medicine and Science, University of Oslo Library, University of Oslo, Oslo, Norway

This supplementary material contains the data that supports the findings of this study.

# of journals	Journal name	ISSN (online); Publisher	Publication date, or update date for author guideline (yyyymmdd or yyyymm if day is not available)	Publication ethics organisation(s) / Is there a particular aspect for which the ethics committee(s) is mentioned, and if so, is there a particular aspect mentioned?	Is there a separate headline/ section/ paragraph for systematic reviews in the author guideline?	Is registration of the protocol of the systematic review in PROSPERO or elsewhere mentioned?	Which methodology guidance is mentioned?	Is there a mention of a reporting standard for systematic reviews? If yes, which standards are mentioned?	Is a procedure to verify the reporting guidelines described?	Is reporting of the search methods for the systematic review mentioned specifically? (for example: number of databases, multiple reviewers, how to report your search strategy, …)	Is the involvement of an information specialist / medical librarian suggested in the author guidelines?	Is depositing / archiving / sharing of search strategies mentioned? (for example: depositing your search strategy on searchRxiv)
1	Acta Paediatrica	ISSN (online): 1651-2227; Publisher: Wiley	202110	COPE, ICMJE / No, the role or commitment is not specified	No	Not mentioned	Not mentioned	No	No	No	No	No
2	Addiction	ISSN (online): 1360-0443; Publisher: Wiley	Not stated	COPE, ICMJE, Other: Group of Editors of Addiction Journals / Yes: Authorship	Yes	Is required/ you must/ you must adhere to.../ should follow/ should be included/ expected to	Not mentioned	Yes: PRISMA (Preferred Reporting Items for Systematic Reviews and Meta-Analyses): Is required/ you must/ you must adhere to.../ should follow/ should be included/ expected to;	Yes. The reporting checklist should be submitted upon submission	Yes: "It is expected that reviews will be 'systematic', which means they will set out very clearly the search strategy (including key words where appropriate),[…]"	No	No
3	Advances in Nutrition	ISSN (online): 2156-5376; Publisher: Oxford University Press	Not stated	COPE, ICMJE / No, the role or commitment is not specified	Systematic reviews are reported as original research/ research article	Is required/ you must/ you must adhere to.../ should follow/ should be included/ expected to	Not mentioned	Yes: PRISMA (Preferred Reporting Items for Systematic Reviews and Meta-Analyses): Recommended/ advised/ suggested/ encouraged/ considered;<br>EQUATOR Network: Recommended/ advised/ suggested/ encouraged/ considered;<br>MOOSE (Meta-analyses of observational studies in epidemiology: Recommended/ advised/ suggested/ encouraged/ considered;	No	No	No	No
4	Aesthetic Plastic Surgery	ISSN (online): 1432-5241; Publisher: Springer Nature	Not stated	COPE / Yes: Authorship	No	Not mentioned	Not mentioned	No	No	No	No	No
5	Ageing Research Reviews	ISSN (online): 1568-1637; Publisher: Elsevier		ICMJE / No, the role or commitment is not specified	No	Not mentioned	Not mentioned	No	No	No	No	No
6	American Journal of Medicine	ISSN (online): 0002-9343; Publisher: Elsevier	Not stated	ICMJE, WAME / Yes: Authorship, Data sharing, Other: WAME: declaration of competing interests	No	Not mentioned	Not mentioned	No	No	No	No	No
7	American Journal of Obstetrics and Gynecology	ISSN (online): 1097-6868; Publisher: Elsevier	Not stated	ICMJE / Yes: Authorship, Data sharing	Yes	Is required/ you must/ you must adhere to.../ should follow/ should be included/ expected to	Not mentioned	Yes: PRISMA (Preferred Reporting Items for Systematic Reviews and Meta-Analyses): Is required/ you must/ you must adhere to.../ should follow/ should be included/ expected to;<br>PRISMA, reference to the 2009 version: is required/ you must/ you must adhere to.../ should follow/ should be included/ expected to;<br>MOOSE (Meta-analyses of observational studies in epidemiology): Is required/ you must/ you must adhere to.../ should follow/ should be included/ expected to	No	No	No	No
8	American Journal of Sports Medicine	ISSN (online): 1552-3365; Publisher: SAGE	Not stated	COPE, ICMJE / No, the role or commitment is not specified	Yes	Not mentioned	Not mentioned	No	No	No	No	No
9	American Journal of therapeutics	ISSN (online): 1536-3686; Publisher: Lippincott Williams & Wilkins/ Wolters Kluwer	Not stated	ICMJE / Yes Other: conflict of interests	Yes	Not mentioned	Not mentioned	No	No	No	No	No
10	Anaesthesia	ISSN (online): 1365-2044; Publisher: Wiley	Not stated	COPE, WAME / Yes: Authorship	Yes	Recommended/ advised/ suggested/ encouraged/ considered	Not mentioned	Yes: PRISMA (Preferred Reporting Items for Systematic Reviews and Meta-Analyses): Is required/ you must/ you must adhere to.../ should follow/ should be included/ expected to;	No	No	No	No
11	Anesthesiology	ISSN (online): 1528-1175; Publisher: Lippincott Williams & Wilkins/ Wolters Kluwer	20220919	COPE, ICMJE, WAME / Yes: Authorship, Other: allegations of misconduct	No	Not mentioned	Not mentioned	Yes: EQUATOR Network: Recommended/ advised/ suggested/ encouraged/ considered	No	No	No	No

# of journals	Journal name	ISSN (online); Publisher	Publication date, or update date for author guideline (yyyymmdd or yyyymm if day is not available)	Publication ethics organisation(s) / Is there a particular aspect for which the ethics committee(s) is mentioned, and if so, is there a particular aspect mentioned?	Is there a separate headline/ section/ paragraph for systematic reviews in the author guideline?	Is registration of the protocol of the systematic review in PROSPERO or elsewhere mentioned?	Which methodology guidance is mentioned?	Is there a mention of a reporting standard for systematic reviews? If yes, which standards are mentioned?	Is a procedure to verify the reporting guidelines described?	Is reporting of the search methods for the systematic review mentioned specifically? (for example: number of databases, multiple reviewers, how to report your search strategy, …)	Is the involvement of an information specialist / medical librarian suggested in the author guidelines?	Is depositing / archiving / sharing of search strategies mentioned? (for example: depositing your search strategy on searchRxiv)
12	Annals of Internal Medicine	ISSN (online): 1539-3704; Publisher: American College of Physicians	20220628	COPE, ICMJE / Yes: Authorship, Other: These sources provide information about issues such as authorship criteria, duplicate publication, scientific misconduct, defining and managing potential conflicts of interest, editorial independence, retraction of publications, and the treatment of research participants.	Yes	Recommended/ advised/ suggested/ encouraged/ considered	Not mentioned	Yes: PRISMA (Preferred Reporting Items for Systematic Reviews and Meta-Analyses): Is required/ you must/ you must adhere to.../ should follow/ should be included/ expected to;<br>EQUATOR Network: Is required/ you must/ you must adhere to.../ should follow/ should be included/ expected to;<br>MOOSE (Meta-analyses of observational studies in epidemiology): Is required/ you must/ you must adhere to.../ should follow/ should be included/ expected to;<br>Other: ENTREQ: Is required/ you must/ you must adhere to.../ should follow/ should be included/ expected to	No	Yes: "Abstract Structure: […] Data Sources (must include start and end search dates) […]"<br>"Text […] Subheadings should be: Data Sources and Searches […]"	No	No
13	Annals of Palliative Medicine	ISSN (online): 2224-5839; Publisher: AME Publishing company	20220905	COPE, ICMJE / No, the role or commitment is not specified	Systematic reviews are reported as original research/ research article	Not mentioned	Cochrane Handbook for Systematic Reviews of Interventions: required/ you must/ you must adhere to.../ should follow/ should be included/ expected to	Yes: PRISMA (Preferred Reporting Items for Systematic Reviews and Meta-Analyses): Is required/ you must/ you must adhere to.../ should follow/ should be included/ expected to;	Yes. The reporting checklist should be submitted upon submission	No	No	No
14	Applied Psychophysiology and Biofeedback	ISSN (online): 1573-3270; Publisher: Springer Nature	Not stated	COPE, ICMJE / Yes: Authorship, Other: acts of misconduct	No	Not mentioned	Not mentioned	Yes: PRISMA (Preferred Reporting Items for Systematic Reviews and Meta-Analyses): Recommended/ advised/ suggested/ encouraged/ considered;<br>PRISMA-P: Recommended/ advised/ suggested/ encouraged/ considered	No	No	No	No
15	Archives of Physical Medicine & Rehabilitation	ISSN (online): 0003-9993; Publisher: Elsevier	20151105	ICMJE / Yes: Authorship, Other: conflict of interests	No	Recommended/ advised/ suggested/ encouraged/ considered	Not mentioned	Yes: PRISMA (Preferred Reporting Items for Systematic Reviews and Meta-Analyses): Is required/ you must/ you must adhere to.../ should follow/ should be included/ expected to;<br>EQUATOR Network: Is required/ you must/ you must adhere to.../ should follow/ should be included/ expected to	Yes. The reporting checklist should be submitted upon submission	Yes: "For review articles, systematic or narrative, readers should be informed of the rationale and details behind the literature search strategy."	No	No
16	Arthroscopy	ISSN (online): 1526-3231; Publisher: Elsevier	202006	ICMJE / Yes: Authorship	Yes	Recommended/ advised/ suggested/ encouraged/ considered	Own methods: "Refer to Harris JD, Brand JC, Cote MP, Dhawan A; Research Pearls: The Significance of Statistics of Perils of Pooling: Pearls and Pitfalls of Meta-analyses and Systematic reviews; Arthroscopy 2017; E-published April 27, 2017 for guidance in design, conduct, reporting, and publishing SR/MA in Arthroscopy.": required/ you must/ you must adhere to.../ should follow/ should be included/ expected to	Yes: PRISMA (Preferred Reporting Items for Systematic Reviews and Meta-Analyses): Is required/ you must/ you must adhere to.../ should follow/ should be included/ expected to;	Other: Submission of a SR should follow the 27-item PRISMA checklist (http://www.prismastatement.org/).	Yes: "Literature Search: The search strategy (terms, string) should be described with enough detail that it could be reproduced. Indicate which databases were searched. Two or more databases should be used (the combination of MEDLINE, EMBASE, and Cochrane will capture 97% of all relevant studies in Orthopedic Surgery SR/MA). The search should be performed independently by two or more study authors to ensure no omission of potentially relevant subjects and resolution of disagreement in the setting of possible study inclusion."	No	No
17	Biomed Research International	ISSN (online): 2314-6141; Publisher: Hindawi	Not stated	COPE, ICMJE, WAME / Yes: Authorship, Other: For more information on COIs, see the guidance from the ICMJE and WAME	Systematic reviews are reported as original research/ research article	Not mentioned	Not mentioned	Yes: PRISMA (Preferred Reporting Items for Systematic Reviews and Meta-Analyses): Recommended/ advised/ suggested/ encouraged/ considered;	Yes. The reporting checklist should be submitted upon submission	No	No	No
18	Bioorganic Chemistry	ISSN (online): 0045-2068; Publisher: Elsevier	Not stated	ICMJE / No, the role or commitment is not specified	No	Not mentioned	Not mentioned	No	No	No	No	No
19	BJOG : an international Journal of Obstetrics and Gynaecology	ISSN (online): 1471-0528; Publisher: Wiley	Not stated	ICMJE / Yes Other: all authors will need to upload a completed ICMJE disclosure of interest form	Yes	Recommended/ advised/ suggested/ encouraged/ considered	Cochrane Handbook for Systematic Reviews of Interventions: required/ you must/ you must adhere to.../ should follow/ should be included/ expected to	Yes: PRISMA (Preferred Reporting Items for Systematic Reviews and Meta-Analyses): Is required/ you must/ you must adhere to.../ should follow/ should be included/ expected to;<br>MOOSE (Meta-analyses of observational studies in epidemiology): Is required/ you must/ you must adhere to.../ should follow/ should be included/ expected to	Yes. The reporting checklist should be submitted upon submission	Yes: "The Search Strategy (described in detail), and tables with the details of the included and excluded studies should be uploaded as online supplementary information only (not to appear in print)."	No	No

# of journals	Journal name	ISSN (online); Publisher	Publication date, or update date for author guideline (yyyymmdd or yyyymm if day is not available)	Publication ethics organisation(s) / Is there a particular aspect for which the ethics committee(s) is mentioned, and if so, is there a particular aspect mentioned?	Is there a separate headline/ section/ paragraph for systematic reviews in the author guideline?	Is registration of the protocol of the systematic review in PROSPERO or elsewhere mentioned?	Which methodology guidance is mentioned?	Is there a mention of a reporting standard for systematic reviews? If yes, which standards are mentioned?	Is a procedure to verify the reporting guidelines described?	Is reporting of the search methods for the systematic review mentioned specifically? (for example: number of databases, multiple reviewers, how to report your search strategy, …)	Is the involvement of an information specialist / medical librarian suggested in the author guidelines?	Is depositing / archiving / sharing of search strategies mentioned? (for example: depositing your search strategy on searchRxiv)
20	Blood	ISSN (online): 1528-0020; Publisher: Washington DC: American Society of Hematology	Not stated	ICMJE / No, the role or commitment is not specified	Systematic reviews are reported as original research/ research article	Not mentioned	Not mentioned	No	No	No	No	No
21	BMC Infectious Diseases	ISSN (online): 1471-2334; Publisher: Springer Nature	Not stated	COPE, ICMJE, WAME / Yes: Authorship, Data sharing	Systematic reviews are reported as original research/ research article	Recommended/ advised/ suggested/ encouraged/ considered	Cochrane Handbook for Systematic Reviews of Interventions: recommended/ advised/ suggested/ encouraged/ considered	Yes: PRISMA (Preferred Reporting Items for Systematic Reviews and Meta-Analyses): Recommended/ advised/ suggested/ encouraged/ considered; PRISMA-P: Recommended/ advised/ suggested/ encouraged/ considered; EQUATOR Network: Recommended/ advised/ suggested/ encouraged/ considered	No	Yes: "Authors of systematic reviews should also provide a link to an additional file from the 'methods' section, which reproduces all details of the search strategy. For an example of how a search strategy should be presented, see the Cochrane Reviewers' Handbook."	No	Yes, Recommended/ advised/ suggested/ encouraged: "Authors are also encouraged to preserve search strings on searchRxiv https://searchrxiv.org/, an archive to support researchers to report, store and share their searches consistently and to enable them to review and re-use existing searches. searchRxiv enables researchers to obtain a digital object identifier (DOI) for their search, allowing it to be cited."
22	BMC Public Health	ISSN (online): 1471-2458; Publisher: Springer Nature	Not stated	COPE, ICMJE / Yes: Authorship, Data sharing	Systematic reviews are reported as original research/ research article	Recommended/ advised/ suggested/ encouraged/ considered	Cochrane Handbook for Systematic Reviews of Interventions: recommended/ advised/ suggested/ encouraged/ considered	Yes: PRISMA (Preferred Reporting Items for Systematic Reviews and Meta-Analyses): Recommended/ advised/ suggested/ encouraged/ considered; PRISMA-P: Recommended/ advised/ suggested/ encouraged/ considered; EQUATOR Network: Recommended/ advised/ suggested/ encouraged/ considered	No	Yes: "Authors of systematic reviews should also provide a link to an additional file from the 'methods' section, which reproduces all details of the search strategy. For an example of how a search strategy should be presented, see the Cochrane Reviewers' Handbook."	No	Yes, Recommended/ advised/ suggested/ encouraged: "Authors are also encouraged to preserve search strings on searchRxiv https://searchrxiv.org/, an archive to support researchers to report, store and share their searches consistently and to enable them to review and re-use existing searches. searchRxiv enables researchers to obtain a digital object identifier (DOI) for their search, allowing it to be cited."
23	BMJ Evidence-Based Medicine	ISSN (online): 2515-4478; Publisher: BMJ	Not stated	COPE, ICMJE, WAME / No, the role or commitment is not specified	Systematic reviews are reported as original research/ research article	Recommended/ advised/ suggested/ encouraged/ considered	Not mentioned	Yes: PRISMA (Preferred Reporting Items for Systematic Reviews and Meta-Analyses): Is required/ you must/ you must adhere to.../ should follow/ should be included/ expected to;	No	No	No	No
24	BMJ Global Health	ISSN (online): 2059-7908; Publisher: BMJ	Not stated	COPE, ICMJE, WAME / No, the role or commitment is not specified	Systematic reviews are reported as original research/ research article	Not mentioned	Not mentioned	No	No	No	No	No
25	BMJ Open	ISSN (online): 2044-6055; Publisher: BMJ	Not stated	ICMJE / Yes: Authorship, Data sharing	Systematic reviews are reported as original research/ research article	Recommended/ advised/ suggested/ encouraged/ considered	Not mentioned	Yes: PRISMA (Preferred Reporting Items for Systematic Reviews and Meta-Analyses): Is required/ you must/ you must adhere to.../ should follow/ should be included/ expected to; PRISMA-P: Is required/ you must/ you must adhere to.../ should follow/ should be included/ expected to; EQUATOR Network: Is required/ you must/ you must adhere to.../ should follow/ should be included/ expected to; MOOSE (Meta-analyses of observational studies in epidemiology): Is required/ you must/ you must adhere to.../ should follow/ should be included/ expected to	Yes. The reporting checklist should be submitted upon submission	No	No	No
26	Brain, Behavior and Immunity	ISSN (online): 1090-2139; Publisher: Elsevier	Not stated	ICMJE, WAME / Yes: Authorship, Data sharing, Peer review	No	Not mentioned	Not mentioned	No	No	No	No	No

# of journals	Journal name	ISSN (online); Publisher	Publication date, or update date for author guideline (yyyymmdd or yyyymm if day is not available)	Publication ethics organisation(s) / Is there a particular aspect for which the ethics committee(s) is mentioned, and if so, is there a particular aspect mentioned?	Is there a separate headline/ section/ paragraph for systematic reviews in the author guideline?	Is registration of the protocol of the systematic review in PROSPERO or elsewhere mentioned?	Which methodology guidance is mentioned?	Is there a mention of a reporting standard for systematic reviews? If yes, which standards are mentioned?	Is a procedure to verify the reporting guidelines described?	Is reporting of the search methods for the systematic review mentioned specifically? (for example: number of databases, multiple reviewers, how to report your search strategy, …)	Is the involvement of an information specialist / medical librarian suggested in the author guidelines?	Is depositing / archiving / sharing of search strategies mentioned? (for example: depositing your search strategy on searchRxiv)
27	British Journal of Psychiatry	ISSN (online): 1472-1465; Publisher: Cambridge University Press	20200415	ICMJE<br>/ Yes: Authorship, Methods	Yes	Is required/ you must/ you must adhere to.../ should follow/ should be included/ expected to	Not mentioned	Yes: PRISMA (Preferred Reporting Items for Systematic Reviews and Meta-Analyses): Is required/ you must/ you must adhere to.../ should follow/ should be included/ expected to;<br>MOOSE (Meta-analyses of observational studies in epidemiology): Is required/ you must/ you must adhere to.../ should follow/ should be included/ expected to	Yes. The reporting checklist should be submitted upon submission	Yes: "Please include a structured abstract […] including data sources [...]"<br>"Supplementary Material - […] Details of a search strategy employed in a literature review [...]"<br>"Review<br>[…] - It is important that the Method section clearly describes the search strategy, study selection criteria and synthesis approach in sufficient detail to ensure the method can be replicated to extract the same data with the same or similar analysis. This should include information about the protocol registration, review software, data sources (bibliographic databases such as PubMed/MEDLINE, Embase, CINAHL, PsycINFO and reference lists from journals or books), MeSH and free text search terms and filters, dates included in the search, screening process, language limitations, inclusion and exclusion criteria, study selection and synthesis approach. To ensure a comprehensive review of the literature, we encourage consideration of publications in non-English languages. Ideally the search should be as current as possible with the search date noted in the manuscript. [...] Supplementary tables, figures and data should include (in this order): 1. PRISMA-P (or equivalent) table 2. Search strings used for various platforms such as MEDLINE, Scopus etc. 3. PICOS table (if relevant) […]"	No	No
28	British Journal of Sports Medicine	ISSN (online): 1473-0480; Publisher: BMJ	Not stated	COPE, ICMJE, WAME<br>/ Yes: Data sharing, Peer review	Yes	Recommended/ advised/ suggested/ encouraged/ considered	Not mentioned	Yes: PRISMA (Preferred Reporting Items for Systematic Reviews and Meta-Analyses): Is required/ you must/ you must adhere to.../ should follow/ should be included/ expected to;<br>Other: PERSiST guidance (implementing PRISMA in Exercise, Rehabilitation, Sport medicine and SporTs science) available at https://bjsm.bmj.com/content/early/2021/10/08/bjsports-2021-103987: Recommended/ advised/ suggested/ encouraged/ considered	Yes. The reporting checklist should be submitted upon submission	Yes: "Systematic reviews provide Level One evidence; they form a critical part of the literature: […] - The literature search should have been completed within 12 months of manuscript submission."	No	No
29	British Medical Journal	ISSN (online): 1756-1833; Publisher: BMJ	Not stated	COPE, ICMJE, WAME<br>/ Yes: Authorship	No	Not mentioned	Not mentioned	No	No	No	No	No
30	Canadian Medical association Journal	ISSN (online): 1488-2329; Publisher: CMAJ Group	Not stated	COPE, ICMJE<br>/ No, the role or commitment is not specified	Yes	Not mentioned	Not mentioned	Yes: PRISMA (Preferred Reporting Items for Systematic Reviews and Meta-Analyses): Is required/ you must/ you must adhere to.../ should follow/ should be included/ expected to;	No	No	No	No
31	Cardiology Journal	ISSN (online): 1898-018X; Publisher: Via Medica	Not stated	COPE, ICMJE<br>/ No, the role or commitment is not specified	No	Not mentioned	Not mentioned	No	No	No	No	No
32	Cardiovascular Research	ISSN (online): 1755-3245; Publisher: Oxford University Press / Oxford Academic	Not stated	COPE, ICMJE<br>/ Yes: Authorship, Data sharing	No	Not mentioned	Not mentioned	Yes: PRISMA (Preferred Reporting Items for Systematic Reviews and Meta-Analyses): Is required/ you must/ you must adhere to.../ should follow/ should be included/ expected to;	No	No	No	No
33	Chemical Senses	ISSN (online): 1464-3553; Publisher: Oxford University Press / Oxford Academic	Not stated	COPE<br>/ Yes: Authorship	No	Not mentioned	Not mentioned	No	No	No	No	No

# of journals	Journal name	ISSN (online); Publisher	Publication date, or update date for author guideline (yyyymmdd or yyyymm if day is not available)	Publication ethics organisation(s) / Is there a particular aspect for which the ethics committee(s) is mentioned, and if so, is there a particular aspect mentioned?	Is there a separate headline/ section/ paragraph for systematic reviews in the author guideline?	Is registration of the protocol of the systematic review in PROSPERO or elsewhere mentioned?	Which methodology guidance is mentioned?	Is there a mention of a reporting standard for systematic reviews? If yes, which standards are mentioned?	Is a procedure to verify the reporting guidelines described?	Is reporting of the search methods for the systematic review mentioned specifically? (for example: number of databases, multiple reviewers, how to report your search strategy, …)	Is the involvement of an information specialist / medical librarian suggested in the author guidelines?	Is depositing / archiving / sharing of search strategies mentioned? (for example: depositing your search strategy on searchRxiv)
34	Chest	ISSN (online): 1931-3543; Publisher: Elsevier	Not stated	ICMJE / No, the role or commitment is not specified	Systematic reviews are reported as original research/ research article	Recommended/ advised/ suggested/ encouraged/ considered	Cochrane Handbook for Systematic Reviews of Interventions: recommended/ advised/ suggested/ encouraged/ considered	Yes: PRISMA (Preferred Reporting Items for Systematic Reviews and Meta-Analyses): Is required/ you must/ you must adhere to.../ should follow/ should be included/ expected to; The Institute of Medicine's Standard for Systematic Reviews: Is required/ you must/ you must adhere to.../ should follow/ should be included/ expected to	Yes. Supplementary file:"Additionally, authors are expected to address all items in the checklist in the writing of the manuscript. PRISMA extension checklist should be submitted as a supplemental file along with other manuscript files"	Yes: "A systematic review involves several steps that can be described in a protocol: […] using several search engines for searches (eg, PubMed-MEDLINE, EMBASE, Scopus, Cochrane library) […]"	No	No
35	Child Development	ISSN (online): 1467-8624; Publisher: Wiley	Not stated	Other: Society for Research in Child Development (SRCD) / No, the role or commitment is not specified	No	Not mentioned	Not mentioned	No	No	No	No	No
36	Circulation	ISSN (online): 1524-4539; Publisher: Lippincott Williams & Wilkins	Not stated	ICMJE / Yes: Authorship, Other: Not for reviews, but for clinical research and trials/contributorship	Systematic reviews are reported as original research/ research article	Not mentioned	Not mentioned	Yes: PRISMA (Preferred Reporting Items for Systematic Reviews and Meta-Analyses): Is required/ you must/ you must adhere to.../ should follow/ should be included/ expected to; MOOSE (Meta-analyses of observational studies in epidemiology): Is required/ you must/ you must adhere to.../ should follow/ should be included/ expected to	Yes. Submission: "Please be prepared to provide the completed appropriate checklist, with page numbers, if requested during submission."	No	No	No
37	Clinical Gastroenterology and Hepatology	ISSN (online): 1542-7714; Publisher: Elsevier	202207	COPE, ICMJE / No, the role or commitment is not specified	Yes	Not mentioned	Not mentioned	Yes: PRISMA (Preferred Reporting Items for Systematic Reviews and Meta-Analyses): Is required/ you must/ you must adhere to.../ should follow/ should be included/ expected to; MOOSE (Meta-analyses of observational studies in epidemiology): Is required/ you must/ you must adhere to.../ should follow/ should be included/ expected to	Yes. The reporting checklist should be submitted upon submission	No	No	No
38	Clinical Infectious Diseases	ISSN (online): 1537-6591; Publisher: Oxford University Press/Oxford Academic	Not stated	COPE, ICMJE / Yes: Authorship, Other: Originality, Conflicts of Interest	No	Not mentioned	Not mentioned	No	No	No	No	No
39	Clinical Microbiology and Infection	ISSN (online): 1469-0691; Publisher: Elsevier	Not stated	ICMJE / Yes: Authorship, Methods, Data sharing, Peer review	Yes	Is required/ you must/ you must adhere to.../ should follow/ should be included/ expected to	Cochrane Handbook for Systematic Reviews of Interventions: recommended/ advised/ suggested/ encouraged/ considered	Yes: PRISMA (Preferred Reporting Items for Systematic Reviews and Meta-Analyses): Is required/ you must/ you must adhere to.../ should follow/ should be included/ expected to;	No	Yes: "Search strategy: Databases searched and search string adapted for each database should be presented (possibly as supplementary material). Study flowchart (in Results) should start transparently from results of described search strategy. Restrictions on study years, publication status or language should be avoided or justified."	No	No
40	Clinical Nutrition	ISSN (online): 1532-1983; Publisher: Elsevier	Not stated	ICMJE / Yes: Authorship, Methods, Data sharing, Peer review	Yes	Not mentioned	Not mentioned	Yes: PRISMA (Preferred Reporting Items for Systematic Reviews and Meta-Analyses): Is required/ you must/ you must adhere to.../ should follow/ should be included/ expected to;	Yes. The reporting checklist should be submitted upon submission	No	No	No
41	Clinical Oral investigations	ISSN (online): 1436-3771; Publisher: Springer Nature	Not stated	COPE, ICMJE / Yes: Authorship	No	Not mentioned	Not mentioned	Yes: PRISMA (Preferred Reporting Items for Systematic Reviews and Meta-Analyses): Recommended/ advised/ suggested/ encouraged/ considered; PRISMA-P: Recommended/ advised/ suggested/ encouraged/ considered; EQUATOR Network: Recommended/ advised/ suggested/ encouraged/ considered	No	No	No	No
42	Clinical Psychology Review	ISSN (online): 1873-7811; Publisher: Elsevier	Not stated	COPE, ICMJE / Yes: Authorship, Methods, Data sharing, Peer review	No	Not mentioned	Not mentioned	No	No	No	No	No

# of journals	Journal name	ISSN (online); Publisher	Publication date, or update date for author guideline (yyyymmdd or yyyymm if day is not available)	Publication ethics organisation(s) / Is there a particular aspect for which the ethics committee(s) is mentioned, and if so, is there a particular aspect mentioned?	Is there a separate headline/ section/ paragraph for systematic reviews in the author guideline?	Is registration of the protocol of the systematic review in PROSPERO or elsewhere mentioned?	Which methodology guidance is mentioned?	Is there a mention of a reporting standard for systematic reviews? If yes, which standards are mentioned?	Is a procedure to verify the reporting guidelines described?	Is reporting of the search methods for the systematic review mentioned specifically? (for example: number of databases, multiple reviewers, how to report your search strategy, …)	Is the involvement of an information specialist / medical librarian suggested in the author guidelines?	Is depositing / archiving / sharing of search strategies mentioned? (for example: depositing your search strategy on searchRxiv)
43	Clinical Rehabilitation	ISSN (online): 1477-0873; Publisher: SAGE	Not stated	COPE, ICMJE / Yes: Authorship, Methods, Data sharing, Peer review	No	Not mentioned	Not mentioned	Yes: PRISMA (Preferred Reporting Items for Systematic Reviews and Meta-Analyses): Is required/ you must/ you must adhere to.../ should follow/ should be included/ expected to; EQUATOR Network: Is required/ you must/ you must adhere to.../ should follow/ should be included/ expected to	Yes. The reporting checklist should be submitted upon submission	No	No	No
44	Cochrane Database of Systematic Reviews	ISSN (online): 1469-493X; Publisher: Wiley	202202	ICMJE / Yes Other: conflict of interests	Yes	Is required/ you must/ you must adhere to.../ should follow/ should be included/ expected to	Cochrane Handbook for Systematic Reviews of Interventions: required/ you must/ you must adhere to.../ should follow/ should be included/ expected to	Yes: MECIR (The Methodological Expectations of Cochrane Intervention Reviews): Is required/ you must/ you must adhere to.../ should follow/ should be included/ expected to	No	Yes: Full section on search methods in the Methodological Expectations of Cochrane Intervention Reviews (MECIR): "1.26 Search methods for identification of studies"	Yes, to assist the authors while creating the search strategy. It is recommended/ advised/ suggested/ encouraged/ should	No
45	Complementary Therapies in Clinical Practice	ISSN (online): 1744-3881; Publisher: Elsevier	Not stated	ICMJE / No, the role or commitment is not specified	No	Not mentioned	Not mentioned	No	No	No	No	No
46	Complementary Therapies in Medicine	ISSN (online): 1873-6963; Publisher: Elsevier	Not stated	ICMJE / No, the role or commitment is not specified	No	Is required/ you must/ you must adhere to.../ should follow/ should be included/ expected to	Not mentioned	Yes: PRISMA (Preferred Reporting Items for Systematic Reviews and Meta-Analyses): Recommended/ advised/ suggested/ encouraged/ considered;	No	No	No	No
47	Critical Care	ISSN (online): 1364-8535; Publisher: BMC/ BioMed Central. Part of Springer Nature.	Not stated	COPE, ICMJE, WAME / No, the role or commitment is not specified	No	Not mentioned	Not mentioned	Yes: PRISMA (Preferred Reporting Items for Systematic Reviews and Meta-Analyses): Recommended/ advised/ suggested/ encouraged/ considered;	No	No	No	Yes, Recommended/ advised/ suggested/ encouraged: "Authors are also encouraged to preserve search strings on searchRxivsearchsearch https://searchrxiv.org/search , an archive to support researchers to report, store and sharesearchtheir searches consistently and to enable them to review and re-use existing searches.searchsearchsearchRxiv enables researchers to obtain a digital object identifier (DOI) for theirsearchsearchsearch, allowing it to be cited."
48	Critical Reviews in Food Science and Nutrition	ISSN (online): 1549-7852; Publisher: Taylor & Francis	20221114	COPE, ICMJE, Other: American Psychological Association (APA) / Yes: Authorship, Methods, Data sharing, Peer review, Other: plagiarism, data fabrication, competing interests	No	Not mentioned	Not mentioned	No	No	No	No	No
49	Critical Reviews in Oncology / Hematology	ISSN (online): 1879-0461; Publisher: Elsevier	Not stated	COPE, ICMJE / Yes: Authorship, Methods, Peer review	No	Not mentioned	Not mentioned	No	No	No	No	No
50	Current Atherosclerosis Reports	ISSN (online): 1534-6242; Publisher: Springer Nature	Not stated	COPE, ICMJE / Yes: Authorship	No	Not mentioned	Not mentioned	Yes: PRISMA (Preferred Reporting Items for Systematic Reviews and Meta-Analyses): Recommended/ advised/ suggested/ encouraged/ considered; PRISMA-P: Recommended/ advised/ suggested/ encouraged/ considered; EQUATOR Network: Recommended/ advised/ suggested/ encouraged/	Other: The above (reporting guidelines) should be summarized in a statement and placed in a 'Declarations' section before the reference list under a heading of 'Ethics approval'.	No	No	No

# of journals	Journal name	ISSN (online); Publisher	Publication date, or update date for author guideline (yyyymmdd or yyyymm if day is not available)	Publication ethics organisation(s) / Is there a particular aspect for which the ethics committee(s) is mentioned, and if so, is there a particular aspect mentioned?	Is there a separate headline/ section/ paragraph for systematic reviews in the author guideline?	Is registration of the protocol of the systematic review in PROSPERO or elsewhere mentioned?	Which methodology guidance is mentioned?	Is there a mention of a reporting standard for systematic reviews? If yes, which standards are mentioned?	Is a procedure to verify the reporting guidelines described?	Is reporting of the search methods for the systematic review mentioned specifically? (for example: number of databases, multiple reviewers, how to report your search strategy, …)	Is the involvement of an information specialist / medical librarian suggested in the author guidelines?	Is depositing / archiving / sharing of search strategies mentioned? (for example: depositing your search strategy on searchRxiv)
51	Current Neurology and Neuroscience Reports	ISSN (online): 1534-6293; Publisher: Springer Nature	Not stated	COPE, ICMJE / Yes: Authorship	No	Not mentioned	Not mentioned	Yes: PRISMA (Preferred Reporting Items for Systematic Reviews and Meta-Analyses): Recommended/ advised/ suggested/ encouraged/ considered; PRISMA-P: Recommended/ advised/ suggested/ encouraged/ considered; EQUATOR Network: Recommended/ advised/ suggested/ encouraged/	Other: The above (reporting guidelines) should be summarized in a statement and placed in a 'Declarations' section before the reference list under a heading of 'Ethics approval'.	No	No	No
52	Current Opinion in Psychology	ISSN (online): 2352-2518; Publisher: Elsevier	Not stated	COPE, ICMJE / Yes: Authorship, Data sharing, Peer review	No	Not mentioned	Not mentioned	No	No	No	No	No
53	Diabetes & Metabolic Syndrome: Clinical Research & Reviews	ISSN (online): 1878-0334; Publisher: Elsevier	Not stated	ICMJE / No, the role or commitment is not specified	No	Not mentioned	Not mentioned	No	No	No	No	No
54	Diabetes, Obesity and Metabolism	ISSN (online): 1463-1326; Publisher: Wiley	20210729	COPE / Yes: Authorship	No	Not mentioned	Not mentioned	Yes: PRISMA (Preferred Reporting Items for Systematic Reviews and Meta-Analyses): Is required/ you must/ you must adhere to.../ should follow/ should be included/ expected to; EQUATOR Network: Is required/ you must/ you must adhere to.../ should follow/ should be included/ expected to	No	No	No	No
55	Disability and Rehabilitation	ISSN (online): 1464-5165; Publisher: Taylor & Francis	20211112	COPE, ICMJE, Other: American Psychological Association (APA) / Yes: Authorship, Methods, Data sharing, Peer review, Other: plagiarism, data fabrication, competing interests	No	Not mentioned	Not mentioned	Yes: PRISMA (Preferred Reporting Items for Systematic Reviews and Meta-Analyses): Recommended/ advised/ suggested/ encouraged/ considered;	No	No	No	No
56	Ecological applications	ISSN (online): 1939-5582; Publisher: Wiley	20220811	Other: Ecological Society of America (ESA) / Yes: Authorship,	No	Not mentioned	Not mentioned	No	No	No	No	No
57	Environmental Research	ISSN (online): 1096-0953; Publisher: Elsevier	Not stated	COPE, ICMJE / Yes: Authorship, Data sharing, Peer review	No	Not mentioned	Not mentioned	No	No	No	No	No
58	Environmental Science and Pollution Research	ISSN (online): 1614-7499; Publisher: Springer Nature	Not stated	COPE, ICMJE / Yes: Authorship,	No	Not mentioned	Not mentioned	No	No	No	No	No
59	European archives of Oto-Rhino-Laryngology	ISSN (online): 1434-4726; Publisher: Springer Nature	Not stated	COPE, ICMJE / Yes: Authorship,	No	Not mentioned	Not mentioned	Yes: PRISMA (Preferred Reporting Items for Systematic Reviews and Meta-Analyses): Recommended/ advised/ suggested/ encouraged/ considered; PRISMA-P: Recommended/ advised/ suggested/ encouraged/ considered; EQUATOR Network: Recommended/ advised/ suggested/ encouraged/	No	No	No	No
60	European Journal of Clinical Microbiology & Infectious Diseases	ISSN (online): 1435-4373; Publisher: Springer Nature	Not stated	COPE, ICMJE / Yes: Authorship,	No	Not mentioned	Not mentioned	Yes: PRISMA (Preferred Reporting Items for Systematic Reviews and Meta-Analyses): Recommended/ advised/ suggested/ encouraged/ considered; PRISMA-P: Recommended/ advised/ suggested/ encouraged/ considered; EQUATOR Network: Recommended/ advised/ suggested/ encouraged/	No	No	No	No
61	European Journal of Epidemiology	ISSN (online): 1573-7284; Publisher: Springer Nature	Not stated	COPE, ICMJE / Yes: Authorship,	No	Not mentioned	Not mentioned	Yes: PRISMA (Preferred Reporting Items for Systematic Reviews and Meta-Analyses): Recommended/ advised/ suggested/ encouraged/ considered; PRISMA-P: Recommended/ advised/ suggested/ encouraged/ considered; EQUATOR Network: Recommended/ advised/ suggested/ encouraged/	No	No	No	No

# of journals	Journal name	ISSN (online); Publisher	Publication date, or update date for author guideline (yyyymmdd or yyyymm if day is not available)	Publication ethics organisation(s) / Is there a particular aspect for which the ethics committee(s) is mentioned, and if so, is there a particular aspect mentioned?	Is there a separate headline/ section/ paragraph for systematic reviews in the author guideline?	Is registration of the protocol of the systematic review in PROSPERO or elsewhere mentioned?	Which methodology guidance is mentioned?	Is there a mention of a reporting standard for systematic reviews? If yes, which standards are mentioned?	Is a procedure to verify the reporting guidelines described?	Is reporting of the search methods for the systematic review mentioned specifically? (for example: number of databases, multiple reviewers, how to report your search strategy, …)	Is the involvement of an information specialist / medical librarian suggested in the author guidelines?	Is depositing / archiving / sharing of search strategies mentioned? (for example: depositing your search strategy on searchRxiv)
62	European Journal of Medical Research	ISSN (online): 2047-783X; Publisher: BMC/ BioMed Central. Part of Springer Nature.	Not stated	COPE, ICMJE, Other: European Medical Writers Association (EMWA) / Yes: Authorship, Methods, Data sharing, Peer review	Systematic reviews are reported as original research/ research article	Not mentioned	Not mentioned	No	No	No	No	Yes, Recommended/ advised/ suggested/ encouraged: "Authors are also encouraged to preserve search strings on searchRxiv https://searchrxiv.org/, an archive to support researchers to report, store and share their searches consistently and to enable them to review and re-use existing searches. searchRxiv enables researchers to obtain a digital object identifier (DOI) for their search, allowing it to be cited."
63	European Journal of Obstetrics, Gynecology, and Reproductive Biology	ISSN (online): 0301-2115; Publisher: Elsevier	Not stated	COPE, ICMJE / Yes: Authorship, Data sharing, Peer review	No	Not mentioned	Not mentioned	No	No	No	No	No
64	European Journal of Surgical Oncology	ISSN (online): 0748-7983; Publisher: Elsevier	Not stated	COPE, ICMJE / Yes: Authorship, Data sharing, Peer review	Systematic reviews are reported as original research/ research article	Not mentioned	Not mentioned	Yes: PRISMA (Preferred Reporting Items for Systematic Reviews and Meta-Analyses): Is required/ you must/ you must adhere to.../ should follow/ should be included/ expected to;	Yes. The reporting checklist should be submitted upon submission	No	No	No
65	European Radiology	ISSN (online): 1432-1084; Publisher: Springer Nature	202201	COPE / Yes: Authorship, Other: Conflicts of Interest, funding	Systematic reviews are reported as original research/ research article	Not mentioned	Not mentioned	Yes: PRISMA (Preferred Reporting Items for Systematic Reviews and Meta-Analyses): Recommended/ advised/ suggested/ encouraged/ considered; EQUATOR Network: Recommended/ advised/ suggested/ encouraged/ considered	No	No	No	No
66	European Review For Medical and Pharmacological Sciences	ISSN (online): 2284-0729; Publisher: Verduci Editore	Not stated	COPE, ICMJE, Other: Council of Science Editors (CSE) / Yes: Authorship, Methods	Yes	Not mentioned	Not mentioned	Yes: PRISMA (Preferred Reporting Items for Systematic Reviews and Meta-Analyses): Is required/ you must/ you must adhere to.../ should follow/ should be included/ expected to;	No	No	No	No
67	European Spine Journal	ISSN (online): 1432-0932; Publisher: Springer Nature	Not stated	COPE, ICMJE / Yes: Authorship, Methods	No	Not mentioned	Not mentioned	Yes: PRISMA (Preferred Reporting Items for Systematic Reviews and Meta-Analyses): Recommended/ advised/ suggested/ encouraged/ considered; PRISMA-P: Recommended/ advised/ suggested/ encouraged/ considered	No	No	No	No
68	Frontiers in Endocrinology	ISSN (online): 1664-2392; Publisher: Frontiers	Not stated	COPE, ICMJE / Yes: Authorship, Methods, Peer review	Yes	Not mentioned	Cochrane Handbook for Systematic Reviews of Interventions; Campbell: required/ you must/ you must adhere to.../ should follow/ should be included/ expected to	Yes: PRISMA (Preferred Reporting Items for Systematic Reviews and Meta-Analyses): Is required/ you must/ you must adhere to.../ should follow/ should be included/ expected to; Campbell Collaboration Guideline: Is required/ you must/ you must adhere to.../ should follow/ should be included/ expected to	No	No	No	No
69	Frontiers in Immunology	ISSN (online): 1664-3224; Publisher: Frontiers	Not stated	COPE, ICMJE / Yes: Authorship, Methods, Peer review	Yes	Not mentioned	Cochrane Handbook for Systematic Reviews of Interventions; Campbell: required/ you must/ you must adhere to.../ should follow/ should be included/ expected to	Yes: PRISMA (Preferred Reporting Items for Systematic Reviews and Meta-Analyses): Is required/ you must/ you must adhere to.../ should follow/ should be included/ expected to; Campbell Collaboration Guideline: Is required/ you must/ you must adhere to.../ should follow/ should be included/ expected to	No	No	No	No
70	Frontiers in Public Health	ISSN (online): 2296-2565; Publisher: Frontiers	Not stated	COPE, ICMJE / Yes: Authorship, Methods, Peer review	Yes	Not mentioned	Cochrane Handbook for Systematic Reviews of Interventions; Campbell: required/ you must/ you must adhere to.../ should follow/ should be included/ expected to	Yes: PRISMA (Preferred Reporting Items for Systematic Reviews and Meta-Analyses): Is required/ you must/ you must adhere to.../ should follow/ should be included/ expected to; Campbell Collaboration Guideline: Is required/ you must/ you must adhere to.../ should follow/ should be included/ expected to	No	No	No	No
71	Gastroenterology	ISSN (online): 1528-0012; Publisher: Elsevier	Not stated	COPE, ICMJE / No, the role or commitment is not specified	Systematic reviews are reported as original research/ research article	Not mentioned	Not mentioned	Yes: PRISMA (Preferred Reporting Items for Systematic Reviews and Meta-Analyses): Is required/ you must/ you must adhere to.../ should follow/ should be included/ expected to; EQUATOR Network: Is required/ you must/ you must adhere to.../ should follow/ should be included/ expected to	No	No	No	No

# of journals	Journal name	ISSN (online); Publisher	Publication date, or update date for author guideline (yyyymmdd or yyyymm if day is not available)	Publication ethics organisation(s) / Is there a particular aspect for which the ethics committee(s) is mentioned, and if so, is there a particular aspect mentioned?	Is there a separate headline/ section/ paragraph for systematic reviews in the author guideline?	Is registration of the protocol of the systematic review in PROSPERO or elsewhere mentioned?	Which methodology guidance is mentioned?	Is there a mention of a reporting standard for systematic reviews? If yes, which standards are mentioned?	Is a procedure to verify the reporting guidelines described?	Is reporting of the search methods for the systematic review mentioned specifically? (for example: number of databases, multiple reviewers, how to report your search strategy, …)	Is the involvement of an information specialist / medical librarian suggested in the author guidelines?	Is depositing / archiving / sharing of search strategies mentioned? (for example: depositing your search strategy on searchRxiv)
72	Globalization and Health	ISSN (online): 1744-8603; Publisher: BMC/ BioMed Central. Part of Springer Nature.	Not stated	COPE, ICMJE, WAME, Other: European Medical Writers Association (EMWA) / Yes: Authorship, Methods, Data sharing, Peer review	No	Not mentioned	Not mentioned	Yes: PRISMA (Preferred Reporting Items for Systematic Reviews and Meta-Analyses): Recommended/ advised/ suggested/ encouraged/ considered; PRISMA-P: Recommended/ advised/ suggested/ encouraged/ considered; EQUATOR Network: Recommended/ advised/ suggested/ encouraged/	No	Yes: "Authors of systematic reviews should also provide a link to an additional file from the 'methods' section, which reproduces all details of the search strategy. For an example of how a search strategy should be presented, see the Cochrane Reviewers' Handbook."	No	No
73	Head & Neck	ISSN (online): 1097-0347; Publisher: Wiley	Not stated	COPE, ICMJE / Yes: Authorship, Methods, Data sharing, Peer review, Other: Conflicts of Interest	No	Not mentioned	Not mentioned	No	No	No	No	No
74	Human Resources For Health	ISSN (online): 1478-4491; Publisher: BMC/ BioMed Central. Part of Springer Nature.	Not stated	COPE, ICMJE, Other: European Medical Writers Association (EMWA) / Yes: Authorship, Methods, Data sharing, Peer review	No	Not mentioned	Not mentioned	Yes: PRISMA (Preferred Reporting Items for Systematic Reviews and Meta-Analyses): Recommended/ advised/ suggested/ encouraged/ considered; PRISMA-P: Recommended/ advised/ suggested/ encouraged/ considered; EQUATOR Network: Recommended/ advised/ suggested/ encouraged/ considered	No	No	No	No
75	indian Journal of Pharmacology	ISSN (online): 1998-3751; Publisher: Lippincott Williams & Wilkins/ Wolters Kluwer	Not stated	COPE, ICMJE, Other: American Psychological Association (APA) / Yes: Authorship, Peer review, Other: Conflicts of Interest, retraction guidelines, plagiarism	No	Not mentioned	Not mentioned	No	No	No	No	No
76	influenza and Other Respiratory Viruses	ISSN (online): 1750-2659; Publisher: Wiley	20200220	COPE, ICMJE / Yes: Authorship,	Yes	Not mentioned	Prospective authors are also strongly encouraged to follow the guidance in the following article: Wille-Jørgensen, P. and Renehan, A. G. (2008), Systematic reviews and meta-analyses in coloproctology: interpretation and potential pitfalls. Colorectal Disease, 10: 21–32. doi: 10.1111/j.1463-1318.2007.01421.x: recommended/ advised/ suggested/ encouraged/ considered	Yes: PRISMA (Preferred Reporting Items for Systematic Reviews and Meta-Analyses): Is required/ you must/ you must adhere to.../ should follow/ should be included/ expected to; QUOROM (Quality of Reporting of Meta-analyses): Recommended/ advised/ suggested/ encouraged/ considered; MOOSE (Meta-analyses of observational studies in epidemiology): Recommended/ advised/ suggested/ encouraged/ considered; Other: STARD (Standards for the Reporting of Diagnostic Accuracy Studies), STROBE-nut (Strengthening the Reporting of Observational Studies in Epidemiology-Nutritional Epidemiology), ARRIVE (Animal Research: Reporting of In Vivo Experiments): Recommended/ advised/ suggested/ encouraged/ considered	Yes. The reporting checklist should be submitted upon submission	Yes: On reporting: "Literature search and study selection: Guidelines encourage a comprehensive description of the literature search such that it is reproducible." And "Yet, SRs of surgical interventions frequently require the inclusion of nonrandomized evidence. One of the first problems encountered is the literature search as standard strategies often miss relevant studies because of uncertainty surrounding the use of appropriate search terms, and in turn, result in the retrieval of a large number of irrelevant records. Methodological filters are available to help minimize this, but have yet to be widely implemented."	No	No
77	Intensive Care Medicine	ISSN (online): 1432-1238; Publisher: Springer Nature	Not stated	COPE, ICMJE / Yes: Authorship, Methods, Peer review	Yes	Not mentioned	Not mentioned	Yes: PRISMA (Preferred Reporting Items for Systematic Reviews and Meta-Analyses): Recommended/ advised/ suggested/ encouraged/ considered; PRISMA-P: Recommended/ advised/ suggested/ encouraged/ considered; EQUATOR Network: Recommended/ advised/ suggested/ encouraged/	No	No	No	No
78	International Journal of audiology	ISSN (online): 1708-8186; Publisher: Taylor & Francis	Not stated	COPE, ICMJE, Other: American Psychological Association (APA) / Yes: Authorship, Methods, Data sharing, Peer revie, Other: plagiarism, data fabrication, competing interests	Yes	Not mentioned	Not mentioned	Yes: PRISMA (Preferred Reporting Items for Systematic Reviews and Meta-Analyses): Recommended/ advised/ suggested/ encouraged/ considered;	Other: It is suggested that systematic reviews be accompanied by a completed Preferred Reporting Items for Systematic Reviews and Meta Analyses (PRISMA) flow diagram checklist.	No	No	No

# of journals	Journal name	ISSN (online); Publisher	Publication date, or update date for author guideline (yyyymmdd or yyyymm if day is not available)	Publication ethics organisation(s) / Is there a particular aspect for which the ethics committee(s) is mentioned, and if so, is there a particular aspect mentioned?	Is there a separate headline/ section/ paragraph for systematic reviews in the author guideline?	Is registration of the protocol of the systematic review in PROSPERO or elsewhere mentioned?	Which methodology guidance is mentioned?	Is there a mention of a reporting standard for systematic reviews? If yes, which standards are mentioned?	Is a procedure to verify the reporting guidelines described?	Is reporting of the search methods for the systematic review mentioned specifically? (for example: number of databases, multiple reviewers, how to report your search strategy, …)	Is the involvement of an information specialist / medical librarian suggested in the author guidelines?	Is depositing / archiving / sharing of search strategies mentioned? (for example: depositing your search strategy on searchRxiv)
79	International Journal of Clinical Practice	ISSN (online): 1742-1241; Publisher: Hindawi | Wiley	Not stated	COPE, ICMJE / Yes: Authorship, Other: plagiarism, conflicts of interest, citation manipulation, fabrication and falsification https://www.hindawi.com/publish-research/authors/publication-ethics/	Systematic reviews are reported as original research/ research article	Recommended/ advised/ suggested/ encouraged/ considered	Not mentioned	Yes: PRISMA (Preferred Reporting Items for Systematic Reviews and Meta-Analyses): Recommended/ advised/ suggested/ encouraged/ considered;	Yes. The reporting checklist should be submitted upon submission	No	No	No
80	International Journal of Colorectal Disease	ISSN (online): 1432-1262; Publisher: Springer Nature	Not stated	COPE, ICMJE / Yes: Authorship, Methods, Peer review	No	Not mentioned	Not mentioned	Yes: PRISMA (Preferred Reporting Items for Systematic Reviews and Meta-Analyses): Recommended/ advised/ suggested/ encouraged/ considered;<br>PRISMA-P: Recommended/ advised/ suggested/ encouraged/ considered;<br>EQUATOR Network: Recommended/ advised/ suggested/ encouraged/	Other: The above (reporting guidelines) should be summarized in a statement and placed in a Declarations' section before the reference list under a heading of 'Ethics approval'.	No	No	No
81	International Journal of Environmental Research and Public Health	ISSN (online): 1660-4601; Publisher: MDPI	Not stated	COPE, ICMJE / Yes: Authorship	No	Not mentioned	Not mentioned	Yes: PRISMA (Preferred Reporting Items for Systematic Reviews and Meta-Analyses): Is required/ you must/ you must adhere to.../ should follow/ should be included/ expected to;	No	No	No	No
82	international Journal of infectious Diseases	ISSN (online): 1201-9712; Publisher: Elsevier	Not stated	COPE, ICMJE / Yes: Authorship, Methods, Data sharing	No	Is required/ you must/ you must adhere to.../ should follow/ should be included/ expected to	Not mentioned	Yes: PRISMA (Preferred Reporting Items for Systematic Reviews and Meta-Analyses): Is required/ you must/ you must adhere to.../ should follow/ should be included/ expected to;	Yes, the reporting checklist should be part of the supplementary materials	No	No	No
83	International Journal of Molecular Sciences	ISSN (online): 1422-0067; Publisher: MDPI	Not stated	COPE, ICMJE / Yes: Authorship,	No	Not mentioned	Not mentioned	Yes: PRISMA (Preferred Reporting Items for Systematic Reviews and Meta-Analyses): Is required/ you must/ you must adhere to.../ should follow/ should be included/ expected to;	Yes, the reporting checklist should be part of the supplementary materials	No	No	No
84	International Journal of Nursing Studies	ISSN (online): 1873-491X; Publisher: Elsevier	Not stated	COPE, ICMJE / Yes: Authorship, Data sharing, Peer review	No	Not mentioned	Not mentioned	Yes: EQUATOR Network: Is required/ you must/ you must adhere to.../ should follow/ should be included/ expected to	Yes. The reporting checklist should be submitted upon submission	No	No	No
85	International Journal of Surgery	ISSN (online): 1743-9159; Publisher: Elsevier	Not stated	COPE, ICMJE / Yes: Authorship, Data sharing, Peer review	Yes	Is required/ you must/ you must adhere to.../ should follow/ should be included/ expected to	Not mentioned	Yes: PRISMA (Preferred Reporting Items for Systematic Reviews and Meta-Analyses): Is required/ you must/ you must adhere to.../ should follow/ should be included/ expected to;	Yes. The reporting checklist should be submitted upon submission	No	No	No
86	International Urogynecology Journal	ISSN (online): 1433-3023; Publisher: Springer Nature	Not stated	COPE / Yes: Authorship,	No	Not mentioned	Not mentioned	No	No	No	No	No
87	JAMA	ISSN (online): 1538-3598; Publisher: JAMA	Not stated	ICMJE / Yes: Authorship,	Yes	Not mentioned	Not mentioned	Yes: PRISMA (Preferred Reporting Items for Systematic Reviews and Meta-Analyses): Is required/ you must/ you must adhere to.../ should follow/ should be included/ expected to;<br>EQUATOR Network: Is required/ you must/ you must adhere to.../ should follow/ should be included/ expected to;<br>MOOSE (Meta-analyses of observational studies in epidemiology): Is required/ you must/ you must adhere to.../ should follow/ should be included/ expected to	Yes. The reporting checklist should be submitted upon submission	Yes: "The search methods should be described in sufficient detail so the search can be reproduced based on the information provided in the manuscript. A summary of the methods of the literature search including this information should be included in the main article; details can be included in an online-only supplement."	Yes, to assist the authors while creating the search strategy. It is recommended/ advised/ suggested/ encouraged/ should	No

# of journals	Journal name	ISSN (online); Publisher	Publication date, or update date for author guideline (yyyymmdd or yyyymm if day is not available)	Publication ethics organisation(s) / Is there a particular aspect for which the ethics committee(s) is mentioned, and if so, is there a particular aspect mentioned?	Is there a separate headline/ section/ paragraph for systematic reviews in the author guideline?	Is registration of the protocol of the systematic review in PROSPERO or elsewhere mentioned?	Which methodology guidance is mentioned?	Is there a mention of a reporting standard for systematic reviews? If yes, which standards are mentioned?	Is a procedure to verify the reporting guidelines described?	Is reporting of the search methods for the systematic review mentioned specifically? (for example: number of databases, multiple reviewers, how to report your search strategy, …)	Is the involvement of an information specialist / medical librarian suggested in the author guidelines?	Is depositing / archiving / sharing of search strategies mentioned? (for example: depositing your search strategy on searchRxiv)
88	JAMA Internal Medicine	ISSN (online): 2168-6114; Publisher: JAMA	20220812	ICMJE / Yes: Authorship,	Yes	Not mentioned	Not mentioned	Yes: PRISMA (Preferred Reporting Items for Systematic Reviews and Meta-Analyses): Is required/ you must/ you must adhere to.../ should follow/ should be included/ expected to; EQUATOR Network: Is required/ you must/ you must adhere to.../ should follow/ should be included/expected to; MOOSE (Meta-analyses of observational studies in epidemiology): Is required/ you must/ you must adhere to.../ should follow/ should be included/ expected to	Yes. The reporting checklist should be submitted upon submission	Yes: "Methods/literature search: The literature search should be as current as possible, ideally with end dates within a month or two before manuscript submission. A search of the primary literature should be conducted, including multiple bibliographic databases (eg, PubMed/MEDLINE, Embase, CINAHL, PsycINFO). This can be facilitated by collaborating with a medical librarian to help with the search. Briefly describe characteristics of the literature searched and included in the review, following the PRISMA reporting guidelines, including the bibliographic databases and other sources searched, search terms used, dates included in the search, date the literature search was conducted, screening process, language limitations, and inclusion and exclusion criteria."	Yes, to assist the authors while creating the search strategy. It is recommended/ advised/ suggested/ encouraged/ should	No
89	JAMA Network Open	ISSN (online): 2574-3805; Publisher: JAMA	20220812	ICMJE / Yes: Authorship,	Yes	Not mentioned	Not mentioned	Yes: PRISMA (Preferred Reporting Items for Systematic Reviews and Meta-Analyses): Is required/ you must/ you must adhere to.../ should follow/ should be included/ expected to; EQUATOR Network: Is required/ you must/ you must adhere to.../ should follow/ should be included/expected to; MOOSE (Meta-analyses of observational studies in epidemiology): Is required/ you must/ you must adhere to.../ should follow/ should be included/ expected to	No	No	No	No
90	JAMA Pediatrics	ISSN (online): 2168-6211; Publisher: JAMA	20220812	ICMJE / Yes: Authorship,	Yes	Not mentioned	Not mentioned	Yes: PRISMA (Preferred Reporting Items for Systematic Reviews and Meta-Analyses): Is required/ you must/ you must adhere to.../ should follow/ should be included/ expected to; EQUATOR Network: Is required/ you must/ you must adhere to.../ should follow/ should be included/expected to; MOOSE (Meta-analyses of observational studies in epidemiology): Is required/ you must/ you must adhere to.../ should follow/ should be included/ expected to	Yes. The reporting checklist should be submitted upon submission	Yes: "Methods/literature search: The literature search should be as current as possible, ideally with end dates within a month or two before manuscript submission. A search of the primary literature should be conducted, including multiple bibliographic databases (eg, PubMed/MEDLINE, Embase, CINAHL, PsycINFO). This can be facilitated by collaborating with a medical librarian to help with the search. Briefly describe characteristics of the literature searched and included in the review, following the PRISMA reporting guidelines, including the bibliographic databases and other sources searched, search terms used, dates included in the search, date the literature search was conducted, screening process, language limitations, and inclusion and exclusion criteria."	Yes, to assist the authors while creating the search strategy. It is recommended/ advised/ suggested/ encouraged/ should	No
91	JAMA Psychiatry	ISSN (online): 2168-6238; Publisher: JAMA	Not stated	ICMJE / Yes: Authorship,	Yes	Not mentioned	Not mentioned	Yes: PRISMA (Preferred Reporting Items for Systematic Reviews and Meta-Analyses): Is required/ you must/ you must adhere to.../ should follow/ should be included/ expected to; EQUATOR Network: Is required/ you must/ you must adhere to.../ should follow/ should be included/expected to; MOOSE (Meta-analyses of observational studies in epidemiology): Is required/ you must/ you must adhere to.../ should follow/ should be included/ expected to	Yes. The reporting checklist should be submitted upon submission	Yes: "Methods/literature search: The literature search should be as current as possible, ideally with end dates within a month or two before manuscript submission. A search of the primary literature should be conducted, including multiple bibliographic databases (eg, PubMed/MEDLINE, Embase, CINAHL, PsycINFO). This can be facilitated by collaborating with a medical librarian to help with the search. Briefly describe characteristics of the literature searched and included in the review, following the PRISMA reporting guidelines, including the bibliographic databases and other sources searched, search terms used, dates included in the search, date the literature search was conducted, screening process, language limitations, and inclusion and exclusion criteria."	Yes, to assist the authors while creating the search strategy. It is recommended/ advised/ suggested/ encouraged/ should	No
92	JBI Evidence Synthesis	ISSN (online): 2689-8381; Publisher: Lippincott Williams & Wilkins/ Wolters Kluwer	Not stated	COPE, ICMJE, WAME / Yes: Authorship,	Yes	Is required/ you must/ you must adhere to.../ should follow/ should be included/ expected to	JBI Manual for Evidence Synthesis: required/ you must/ you must adhere to.../ should follow/ should be included/ expected to	Yes: PRISMA (Preferred Reporting Items for Systematic Reviews and Meta-Analyses): Is required/ you must/ you must adhere to.../ should follow/ should be included/ expected to; PRISMA-P: Recommended/ advised/ suggested/ encouraged/ considered	Yes. The reporting checklist should be submitted upon submission	No	No	No
93	Journal of Advanced Nursing	ISSN (online): 1365-2648; Publisher: Wiley	Not stated	COPE, ICMJE / Yes: Authorship,	Yes	Is required/ you must/ you must adhere to.../ should follow/ should be included/ expected to	Cochrane Handbook for Systematic Reviews of Interventions; and JBI Manual for Evidence Synthesis; and COSMIN: Systematic reviews and meta-analyses of tools and instruments. Must follow COSMIN guidelines and standards: required/ you must/ you must adhere to.../ should follow/ should be included/ expected to	Yes: PRISMA (Preferred Reporting Items for Systematic Reviews and Meta-Analyses): Is required/ you must/ you must adhere to.../ should follow/ should be included/ expected to; EQUATOR Network: Is required/ you must/ you must adhere to.../ should follow/ should be included/expected to; Other: PAGER Framework (scoping reviews), Synthesis Without Meta-analysis (SWiM) guideline: Is required/ you must/ you must adhere to.../ should follow/ should be included/ expected to	No	No	No	No
94	Journal of Affective Disorders	ISSN (online): 1573-2517; Publisher: Elsevier	Not stated	ICMJE / Yes: Authorship,	No	Not mentioned	Not mentioned	No	No	No	No	No

# of journals	Journal name	ISSN (online); Publisher	Publication date, or update date for author guideline (yyyymmdd or yyyymm if day is not available)	Publication ethics organisation(s) / Is there a particular aspect for which the ethics committee(s) is mentioned, and if so, is there a particular aspect mentioned?	Is there a separate headline/ section/ paragraph for systematic reviews in the author guideline?	Is registration of the protocol of the systematic review in PROSPERO or elsewhere mentioned?	Which methodology guidance is mentioned?	Is there a mention of a reporting standard for systematic reviews? If yes, which standards are mentioned?	Is a procedure to verify the reporting guidelines described?	Is reporting of the search methods for the systematic review mentioned specifically? (for example: number of databases, multiple reviewers, how to report your search strategy, …)	Is the involvement of an information specialist / medical librarian suggested in the author guidelines?	Is depositing / archiving / sharing of search strategies mentioned? (for example: depositing your search strategy on searchRxiv)
95	Journal of Antibiotics	ISSN (online): 1881-1469; Publisher: Springer Nature	20220616	/	No	Not mentioned	Not mentioned	No	No	No	No	No
96	Journal of Clinical Virology	ISSN (online): 1873-5967; Publisher: Elsevier	Not stated	COPE, ICMJE / Yes: Authorship, Data sharing, Peer review	No	Not mentioned	Not mentioned	No	No	No	No	No
97	Journal of Critical Care	ISSN (online): 1557-8615; Publisher: Elsevier	Not stated	ICMJE / No, the role or commitment is not specified	Systematic reviews are reported as original research/ research article	Not mentioned	Not mentioned	No	No	No	No	No
98	Journal of Evidence Based Medicine	ISSN (online): 1756-5391; Publisher: Wiley	20140806	COPE, ICMJE / No, the role or commitment is not specified	No	Not mentioned	Not mentioned	Yes: PRISMA (Preferred Reporting Items for Systematic Reviews and Meta-Analyses): Recommended/ advised/ suggested/ encouraged/ considered;	No	No	No	No
99	Journal of Global Health	ISSN (online): 2047-2986; Publisher: International Society of Global Health (ISoGH)	Not stated	COPE, ICMJE / Yes: Authorship, Peer review, Other: Research integrity The Editorial Board of JoGH is devoted to the promotion of scientific integrity as a vital component of the research process. JoGH follows the ethics flowcharts developed by the Committee on Publication Ethics (COPE) for dealing with cases of possible misconduct. The COPE flowcharts are available here .	Systematic reviews are reported as original research/ research article	Not mentioned	Not mentioned	Yes: PRISMA (Preferred Reporting Items for Systematic Reviews and Meta-Analyses): Is required/ you must/ you must adhere to.../ should follow/ should be included/ expected to;<br>EQUATOR Network: Is required/ you must/ you must adhere to.../ should follow/ should be inc	No	No	No	No
100	Journal of Hazardous Materials	ISSN (online): 1873-3336; Publisher: Elsevier	Not stated	COPE, ICMJE / Yes: Authorship, Methods, Data sharing	No	Not mentioned	Not mentioned	No	No	No	No	No
101	Journal of Hepatology	ISSN (online): 1600-0641; Publisher: Elsevier	Not stated	COPE, ICMJE / Yes: Authorship, Methods, Data sharing	No	Not mentioned	Not mentioned	Yes: PRISMA (Preferred Reporting Items for Systematic Reviews and Meta-Analyses): Is required/ you must/ you must adhere to.../ should follow/ should be included/ expected to;<br>PRISMA-P: Is required/ you must/ you must adhere to.../ should follow/ should be included/ expected to;<br>EQUATOR Network: Is required/ you must/ you must adhere to.../ should follow/ should be included/ expected to;<br>MOOSE (Meta-analyses of observational studies in epidemiology): Is required/ you must/ you must adhere to.../ should follow/ should be included/ expected to	No	No	No	No
102	Journal of Infection	ISSN (online): 1532-2742; Publisher: Elsevier	Not stated	COPE, ICMJE / Yes: Authorship, Methods, Data sharing	No	Not mentioned	Not mentioned	No	No	No	No	No
103	Journal of Infectious Diseases	ISSN (online): 1537-6613; Publisher: Oxford University Press / Oxford Academic	Not stated	Not stated	No	Not mentioned	Not mentioned	No	No	No	No	No
104	Journal of Maternal-Fetal & Neonatal Medicine	ISSN (online): 1476-4954; Publisher: Taylor & Francis	20220915	ICMJE, Other: Taylor & Francis Editorial Policies / Yes: Authorship, Methods	Yes	Not mentioned	Not mentioned	Yes: PRISMA (Preferred Reporting Items for Systematic Reviews and Meta-Analyses): Is required/ you must/ you must adhere to.../ should follow/ should be included/ expected to;<br>PRISMA-P: Is required/ you must/ you must adhere to.../ should follow/ should be included/ expected to	Yes. The reporting checklist should be submitted upon submission	No	No	No

# of journals	Journal name	ISSN (online); Publisher	Publication date, or update date for author guideline (yyyymmdd or yyyymm if day is not available)	Publication ethics organisation(s) / Is there a particular aspect for which the ethics committee(s) is mentioned, and if so, is there a particular aspect mentioned?	Is there a separate headline/ section/ paragraph for systematic reviews in the author guideline?	Is registration of the protocol of the systematic review in PROSPERO or elsewhere mentioned?	Which methodology guidance is mentioned?	Is there a mention of a reporting standard for systematic reviews? If yes, which standards are mentioned?	Is a procedure to verify the reporting guidelines described?	Is reporting of the search methods for the systematic review mentioned specifically? (for example: number of databases, multiple reviewers, how to report your search strategy, …)	Is the involvement of an information specialist / medical librarian suggested in the author guidelines?	Is depositing / archiving / sharing of search strategies mentioned? (for example: depositing your search strategy on searchRxiv)
105	Journal of Medical Internet Research	ISSN (online): 1438-8871; Publisher: JMIR Publications	Not stated	ICMJE / No, the role or commitment is not specified	No	Not mentioned	Not mentioned	Yes: PRISMA (Preferred Reporting Items for Systematic Reviews and Meta-Analyses): Is required/ you must/ you must adhere to.../ should follow/ should be included/ expected to; EQUATOR Network: Is required/ you must/ you must adhere to.../ should follow/ should be inc	Yes. Supplementary file: "We strongly recommend to upload completed checklists as supplementary files when submitting your JMIR manuscript."	No	No	No
106	Journal of Medical Virology	ISSN (online): 1096-9071; Publisher: Wiley	Not stated	Not stated	No	Not mentioned	Not mentioned	No	No	No	No	Yes, Required/ must/ should: "Wiley Data Sharing Polices Journal of Medical Virology expects that data supporting the results in the paper will be archived in an appropriate public repository. Authors are required to provide a data availability statement to describe the availability or the absence of shared data. When data have been shared, authors are required to include in their data availability statement a link to the repository they have used, and to cite the data they have shared. Whenever possible the scripts and other artefacts used to generate the analyses presented in the paper should also be publicly archived. If sharing data compromises ethical standards or legal requirements then authors are not expected to share it. Please see our Wiley Data Sharing Polices page for more information."
107	Journal of Microbiology Immunology and infection	ISSN (online): 1684-1182; Publisher: Elsevier	Not stated	ICMJE / No, the role or commitment is not specified	No	Not mentioned	Not mentioned	No	No	No	No	No
108	Journal of Neurology	ISSN (online): 1432-1459; Publisher: Springer Nature	Not stated	COPE, ICMJE / Yes: Authorship,	No	Not mentioned	Not mentioned	Yes: PRISMA (Preferred Reporting Items for Systematic Reviews and Meta-Analyses): Recommended/ advised/ suggested/ encouraged/ considered; PRISMA-P: Recommended/ advised/ suggested/ encouraged/ considered; EQUATOR Network: Recommended/ advised/ suggested/ encouraged/	No	No	No	No
109	Journal of Neurology, Neurosurgery and Psychiatry	ISSN (online): 1468-330X; Publisher: BMJ	Not stated	COPE / Yes: Peer review	Yes	Not mentioned	Not mentioned	No	No	No	No	No
110	Journal of Orthopaedic Surgery and Research	ISSN (online): 1749-799X; Publisher: BMC/ BioMed Central. Part of Springer Nature.	Not stated	COPE, ICMJE, WAME / No, the role or commitment is not specified	Yes	Recommended/ advised/ suggested/ encouraged/ considered	Not mentioned	Yes: PRISMA (Preferred Reporting Items for Systematic Reviews and Meta-Analyses): Is required/ you must/ you must adhere to.../ should follow/ should be included/ expected to;	Yes. The reporting checklist should be submitted upon submission	No	No	Yes, Recommended/ advised/ suggested/ encouraged:"Authors are also encouraged to preserve search strings on searchRxiv https://searchrxiv.org/, an archive to support researchers to report, store and share their searches consistently and to enable them to review and re-use existing searches. searchRxiv enables researchers to obtain a digital object identifier (DOI) for their search, allowing it to be cited."
111	Journal of Plastic, Reconstructive & Aesthetic Surgery	ISSN (online): 1878-0539; Publisher: Elsevier	Not stated	COPE, ICMJE / No, the role or commitment is not specified	No	Not mentioned	Not mentioned	Yes: PRISMA (Preferred Reporting Items for Systematic Reviews and Meta-Analyses): Is required/ you must/ you must adhere to.../ should follow/ should be included/ expected to;	No	No	No	No
112	Journal of Prosthetic Dentistry	ISSN (online): 0022-3913; Publisher: Elsevier	Not stated	ICMJE / No, the role or commitment is not specified	Yes	Not mentioned	Cochrane Handbook for Systematic Reviews of Interventions: recommended/ advised/ suggested/ encouraged/ considered	No	No	No	No	No

# of journals	Journal name	ISSN (online); Publisher	Publication date, or update date for author guideline (yyyymmdd or yyyymm if day is not available)	Publication ethics organisation(s) / Is there a particular aspect for which the ethics committee(s) is mentioned, and if so, is there a particular aspect mentioned?	Is there a separate headline/ section/ paragraph for systematic reviews in the author guideline?	Is registration of the protocol of the systematic review in PROSPERO or elsewhere mentioned?	Which methodology guidance is mentioned?	Is there a mention of a reporting standard for systematic reviews? If yes, which standards are mentioned?	Is a procedure to verify the reporting guidelines described?	Is reporting of the search methods for the systematic review mentioned specifically? (for example: number of databases, multiple reviewers, how to report your search strategy, …)	Is the involvement of an information specialist / medical librarian suggested in the author guidelines?	Is depositing / archiving / sharing of search strategies mentioned? (for example: depositing your search strategy on searchRxiv)
113	Journal of Shoulder and Elbow Surgery	ISSN (online): 1532-6500; Publisher: Elsevier	Not stated	ICMJE / No, the role or commitment is not specified	Yes	Not mentioned	Not mentioned	Yes: PRISMA (Preferred Reporting Items for Systematic Reviews and Meta-Analyses): Is required/ you must/ you must adhere to.../ should follow/ should be included/ expected to;	No	No	No	No
114	Journal of the American Academy of Child and Adolescent Psychiatry	ISSN (online): 1527-5418; Publisher: Elsevier	Not stated	COPE, ICMJE / Yes: Authorship,	Yes	Recommended/ advised/ suggested/ encouraged/ considered	Not mentioned	Yes: PRISMA (Preferred Reporting Items for Systematic Reviews and Meta-Analyses): Is required/ you must/ you must adhere to.../ should follow/ should be included/ expected to;	Yes, the reporting checklist should be part of the supplementary materials	Yes: "Review articles should provide a critical assessment of the literature and include the search and selection criteria for data sources."	No	No
115	Journal of the American Academy of Dermatology	ISSN (online): 1097-6787; Publisher: Elsevier	Not stated	COPE, ICMJE, WAME / No, the role or commitment is not specified	Yes	Is required/ you must/ you must adhere to.../ should follow/ should be included/ expected to	Not mentioned	Yes: PRISMA (Preferred Reporting Items for Systematic Reviews and Meta-Analyses): Is required/ you must/ you must adhere to.../ should follow/ should be included/ expected to; MOOSE (Meta-analyses of observational studies in epidemiology): Is required/ you must/ you must adhere to.../ should follow/ should be included/ expected to	Yes. The reporting checklist should be submitted upon submission	No	No	No
116	Journal of the Renin-angiotensin-aldosterone System	ISSN (online): 1752-8976; Publisher: SAGE	Not stated	COPE, ICMJE / No, the role or commitment is not specified	No	Not mentioned	Not mentioned	Yes: EQUATOR Network: Is required/ you must/ you must adhere to.../ should follow/ should be included/ expected to	Yes. The reporting checklist should be submitted upon submission	No	No	No
117	The Journal of Urology	ISSN (online): 1527-3792; Publisher: Lippincott Williams & Wilkins/ Wolters Kluwer	Not stated	COPE, ICMJE / Yes: Authorship, Data sharing, Other: plagiarism, patient consent	No	Not mentioned	Not mentioned	Yes: EQUATOR Network: Is required/ you must/ you must adhere to.../ should follow/ should be included/ expected to; Other: For statistical guidelines, please see "Guidelines for Reporting of Statistics for Clinical Research in Urology" (https://www.auajournals.org/doi/10.1097/JU.0000000000000001). For guidelines on tables and figures, please see "Guidelines for Reporting of Figures and Tables for Clinical Research in Urology" (https://www.auajournals.org/doi/full/10.1097/JU.0000000000001096). and Reporting Standards: At The Journal of Urology!, we expect authors to follow well-defined reporting standards to improve the reliability and value of published health research and promote transparent and accurate reporting. These standards can be found at EQUATOR Network network (https:// www.EQUATOR Network-network.org). We also advocate for authors to follow best practices for reporting of adverse events such as Common Terminology Criteria for Adverse Events (CTCAE), which is an internationally accepted standard for defining and categorizing adverse events.: Is required/ you must/ you must adhere to.../ should follow/ should be included/ expected to	No	No	No	No
118	Knee Surgery, Sports Traumatology, Arthroscopy	ISSN (online): 1433-7347; Publisher: Springer Nature	Not stated	COPE, ICMJE / Yes: Authorship,	No	Not mentioned	Not mentioned	Yes: PRISMA (Preferred Reporting Items for Systematic Reviews and Meta-Analyses): Is required/ you must/ you must adhere to.../ should follow/ should be included/ expected to; PRISMA-P: Is required/ you must/ you must adhere to.../ should follow/ should be included/ expected to	No	No	No	No
119	Lancet	ISSN (online): 1474-547X; Publisher: Elsevier	Not stated	COPE, ICMJE / Yes: Authorship, Methods, Data sharing	Yes	Is required/ you must/ you must adhere to.../ should follow/ should be included/ expected to	There is clear methodology guidance and instructions from Lancet in a separte file on their website: required/ you must/ you must adhere to.../ should follow/ should be included/ expected to	Yes: PRISMA (Preferred Reporting Items for Systematic Reviews and Meta-Analyses): Is required/ you must/ you must adhere to.../ should follow/ should be included/ expected to; EQUATOR Network: Is required/ you must/ you must adhere to.../ should follow/ should be inc	No	Yes: "Methods - Search strategy and selection criteria: Describe the data sources assessed. List databases searched and exact date cutoffs. Provide search terms used for at least one database such that the search could be repeated."	No	Yes, Recommended/ advised/ suggested/ encouraged:"supplementary file: For Lancet-group systematic reviews we recommend you include these items in the appendix. • Full search terms for one database."
120	Lancet Child & Adolescent Health	ISSN (online): 2352-4650; Publisher: Elsevier	Not stated	COPE, ICMJE / Yes: Authorship,	Yes	Is required/ you must/ you must adhere to.../ should follow/ should be included/ expected to	The Lancet's formatting guidelines for systematic reviews and meta-analyses: required/ you must/ you must adhere to.../ should follow/ should be included/ expected to	Yes: PRISMA (Preferred Reporting Items for Systematic Reviews and Meta-Analyses): Is required/ you must/ you must adhere to.../ should follow/ should be included/ expected to; EQUATOR Network: Is required/ you must/ you must adhere to.../ should follow/ should be inc	No	Yes: "Methods - Search strategy and selection criteria: Describe the data sources assessed. List databases searched and exact date cutoffs. Provide search terms used for at least one database such that the search could be repeated."	No	Yes, Recommended/ advised/ suggested/ encouraged:"supplementary file: For Lancet-group systematic reviews we recommend you include these items in the appendix. • Full search terms for one database."

# of journals	Journal name	ISSN (online); Publisher	Publication date, or update date for author guideline (yyyymmdd or yyyymm if day is not available)	Publication ethics organisation(s) / Is there a particular aspect for which the ethics committee(s) is mentioned, and if so, is there a particular aspect mentioned?	Is there a separate headline/ section/ paragraph for systematic reviews in the author guideline?	Is registration of the protocol of the systematic review in PROSPERO or elsewhere mentioned?	Which methodology guidance is mentioned?	Is there a mention of a reporting standard for systematic reviews? If yes, which standards are mentioned?	Is a procedure to verify the reporting guidelines described?	Is reporting of the search methods for the systematic review mentioned specifically? (for example: number of databases, multiple reviewers, how to report your search strategy, …)	Is the involvement of an information specialist / medical librarian suggested in the author guidelines?	Is depositing / archiving / sharing of search strategies mentioned? (for example: depositing your search strategy on searchRxiv)
121	Lancet Digital Health	ISSN (online): 2589-7500; Publisher: Elsevier	Not stated	COPE, ICMJE / Yes: Authorship, Data sharing	Yes	Is required/ you must/ you must adhere to.../ should follow/ should be included/ expected to	The Lancet's formatting guidelines for systematic reviews and meta-analyses: required/ you must/ you must adhere to.../ should follow/ should be included/ expected to	Yes: PRISMA (Preferred Reporting Items for Systematic Reviews and Meta-Analyses): Is required/ you must/ you must adhere to.../ should follow/ should be included/ expected to; EQUATOR Network: Is required/ you must/ you must adhere to.../ should follow/ should be inc	No	Yes: "Methods - Search strategy and selection criteria: Describe the data sources assessed. List databases searched and exact date cutoffs. Provide search terms used for at least one database such that the search could be repeated."	No	Yes, Recommended/ advised/ suggested/ encouraged:"supplementary file: For Lancet-group systematic reviews we recommend you include these items in the appendix. • Full search terms for one database."
122	Lancet Gastroenterology & Hepatology	ISSN (online): 2468-1156; Publisher: Elsevier	Not stated	COPE, ICMJE / Yes: Authorship, Data sharing	Yes	Is required/ you must/ you must adhere to.../ should follow/ should be included/ expected to	The Lancet's formatting guidelines for systematic reviews and meta-analyses: required/ you must/ you must adhere to.../ should follow/ should be included/ expected to	Yes: PRISMA (Preferred Reporting Items for Systematic Reviews and Meta-Analyses): Is required/ you must/ you must adhere to.../ should follow/ should be included/ expected to; EQUATOR Network: Is required/ you must/ you must adhere to.../ should follow/ should be inc	No	Yes: "Methods - Search strategy and selection criteria: Describe the data sources assessed. List databases searched and exact date cutoffs. Provide search terms used for at least one database such that the search could be repeated."	No	Yes, Recommended/ advised/ suggested/ encouraged:"supplementary file: For Lancet-group systematic reviews we recommend you include these items in the appendix. • Full search terms for one database."
123	Lancet Global Health	ISSN (online): 2214-109X; Publisher: Elsevier	Not stated	COPE, ICMJE / Yes: Authorship,	Yes	Is required/ you must/ you must adhere to.../ should follow/ should be included/ expected to	The Lancet's formatting guidelines for systematic reviews and meta-analyses: required/ you must/ you must adhere to.../ should follow/ should be included/ expected to	Yes: PRISMA (Preferred Reporting Items for Systematic Reviews and Meta-Analyses): Is required/ you must/ you must adhere to.../ should follow/ should be included/ expected to; EQUATOR Network: Is required/ you must/ you must adhere to.../ should follow/ should be inc	No	Yes: "Methods - Search strategy and selection criteria: Describe the data sources assessed. List databases searched and exact date cutoffs. Provide search terms used for at least one database such that the search could be repeated."	No	Yes, Recommended/ advised/ suggested/ encouraged:"supplementary file: For Lancet-group systematic reviews we recommend you include these items in the appendix. • Full search terms for one database."
124	Lancet Microbe	ISSN (online): 2666-5247; Publisher: Elsevier	Not stated	COPE, ICMJE / Yes: Authorship,	Yes	Is required/ you must/ you must adhere to.../ should follow/ should be included/ expected to	The Lancet's formatting guidelines for systematic reviews and meta-analyses: required/ you must/ you must adhere to.../ should follow/ should be included/ expected to	Yes: PRISMA (Preferred Reporting Items for Systematic Reviews and Meta-Analyses): Is required/ you must/ you must adhere to.../ should follow/ should be included/ expected to; EQUATOR Network: Is required/ you must/ you must adhere to.../ should follow/ should be inc	No	Yes: "Methods - Search strategy and selection criteria: Describe the data sources assessed. List databases searched and exact date cutoffs. Provide search terms used for at least one database such that the search could be repeated."	No	Yes, Recommended/ advised/ suggested/ encouraged:"supplementary file: For Lancet-group systematic reviews we recommend you include these items in the appendix. • Full search terms for one database."
125	Lancet Psychiatry	ISSN (online): 2215-0374; Publisher: Elsevier	Not stated	COPE, ICMJE / Yes: Authorship,	Yes	Is required/ you must/ you must adhere to.../ should follow/ should be included/ expected to	The Lancet's formatting guidelines for systematic reviews and meta-analyses: required/ you must/ you must adhere to.../ should follow/ should be included/ expected to	Yes: PRISMA (Preferred Reporting Items for Systematic Reviews and Meta-Analyses): Is required/ you must/ you must adhere to.../ should follow/ should be included/ expected to; EQUATOR Network: Is required/ you must/ you must adhere to.../ should follow/ should be inc	No	Yes: "Methods - Search strategy and selection criteria: Describe the data sources assessed. List databases searched and exact date cutoffs. Provide search terms used for at least one database such that the search could be repeated."	No	Yes, Recommended/ advised/ suggested/ encouraged:"supplementary file: For Lancet-group systematic reviews we recommend you include these items in the appendix. • Full search terms for one database."
126	Lancet Respiratory Medicine	ISSN (online): 2213-2619; Publisher: Elsevier	Not stated	COPE, ICMJE / Yes: Authorship,	Yes	Is required/ you must/ you must adhere to.../ should follow/ should be included/ expected to	The Lancet's formatting guidelines for systematic reviews and meta-analyses: required/ you must/ you must adhere to.../ should follow/ should be included/ expected to	Yes: PRISMA (Preferred Reporting Items for Systematic Reviews and Meta-Analyses): Is required/ you must/ you must adhere to.../ should follow/ should be included/ expected to; EQUATOR Network: Is required/ you must/ you must adhere to.../ should follow/ should be inc	No	Yes: "Methods - Search strategy and selection criteria: Describe the data sources assessed. List databases searched and exact date cutoffs. Provide search terms used for at least one database such that the search could be repeated."	No	Yes, Recommended/ advised/ suggested/ encouraged:"supplementary file: For Lancet-group systematic reviews we recommend you include these items in the appendix. • Full search terms for one database."
127	Laryngoscope	ISSN (online): 1531-4995; Publisher: Wiley	20170523	COPE, ICMJE / Yes: Authorship,	Yes	Not mentioned	Not mentioned	Yes: PRISMA (Preferred Reporting Items for Systematic Reviews and Meta-Analyses): Is required/ you must/ you must adhere to.../ should follow/ should be included/ expected to; MOOSE (Meta-analyses of observational studies in epidemiology): Is required/ you must/ you must adhere to.../ should follow/ should be included/ expected to; Prisma extensions are mentioned	No	Yes: "In brief, Systematic Reviews should include the following: […] - Explicit description of the electronic search strategy and databases used (at least 3), including Mesh titles, dates of inclusion, and the names of those performing the search Report of the results of the search, the studies screened, and the studies included […]."	Yes, to assist the authors while creating the search strategy. It is recommended/ advised/ suggested/ encouraged/ should	No
128	Mayo Clinic Proceedings	ISSN (online): 1942-5546; Publisher: Elsevier	Not stated	ICMJE / Yes: Authorship, Other: Conflicts of Interest	Yes	Not mentioned	Not mentioned	Yes: PRISMA (Preferred Reporting Items for Systematic Reviews and Meta-Analyses): Is required/ you must/ you must adhere to.../ should follow/ should be included/ expected to; EQUATOR Network: Is required/ you must/ you must adhere to.../ should follow/ should be included/expected to	Yes. The reporting checklist should be submitted upon submission	Yes: "Authors are strongly encouraged to describe within the abstract and manuscript text the methods used to focus their search of the literature (eg, PubMed, MEDLINE), the search terms used, and the date limitations of the search."	No	No
129	Medicina	ISSN (online): 1648-9144; Publisher: MDPI	Not stated	COPE, ICMJE / No, the role or commitment is not specified	No	Not mentioned	Not mentioned	Yes: PRISMA (Preferred Reporting Items for Systematic Reviews and Meta-Analyses): Is required/ you must/ you must adhere to.../ should follow/ should be included/ expected to;	No	No	No	No
130	Medicine	ISSN (online): 1536-5964; Publisher: Lippincott Williams & Wilkins/ Wolters Kluwer	Not stated	COPE, ICMJE, WAME / Yes: Authorship, Other: plagiarism	Yes	Not mentioned	Not mentioned	Yes: PRISMA (Preferred Reporting Items for Systematic Reviews and Meta-Analyses): Is required/ you must/ you must adhere to.../ should follow/ should be included/ expected to; PRISMA-P: Is required/ you must/ you must adhere to.../ should follow/ should be included/ expected to; EQUATOR Network: Is required/ you must/ you must adhere to.../ should follow/ should be included/ expected to	Yes. The reporting checklist should be submitted upon submission	No	No	No

# of journals	Journal name	ISSN (online); Publisher	Publication date, or update date for author guideline (yyyymmdd or yyyymm if day is not available)	Publication ethics organisation(s) / Is there a particular aspect for which the ethics committee(s) is mentioned, and if so, is there a particular aspect mentioned?	Is there a separate headline/ section/ paragraph for systematic reviews in the author guideline?	Is registration of the protocol of the systematic review in PROSPERO or elsewhere mentioned?	Which methodology guidance is mentioned?	Is there a mention of a reporting standard for systematic reviews? If yes, which standards are mentioned?	Is a procedure to verify the reporting guidelines described?	Is reporting of the search methods for the systematic review mentioned specifically? (for example: number of databases, multiple reviewers, how to report your search strategy, …)	Is the involvement of an information specialist / medical librarian suggested in the author guidelines?	Is depositing / archiving / sharing of search strategies mentioned? (for example: depositing your search strategy on searchRxiv)
131	Modern Pathology	ISSN (online): 1530-0285; Publisher: Springer Nature	Not stated	ICMJE / No, the role or commitment is not specified	No	Not mentioned	Not mentioned	No	No	No	No	Yes, Required/ must/ should:"Data Availability Statement: All manuscripts must include an 'Data availability' statement. Data availability statements should provide information on where data supporting the results reported in the article can be found including, where applicable, hyperlinks to publicly archived datasets analyzed or generated during the study. Examples of suitable statements include:"
132	Nature Communications	ISSN (online): 2041-1723; Publisher: Springer Nature	Not stated	Not stated /	No	Not mentioned	Not mentioned	No	Yes. The reporting checklist should be submitted upon submission	No	No	No
133	Nature Human Behaviour	ISSN (online): 2397-3374; Publisher: Springer Nature	Not stated	Not stated /	No	Not mentioned	Not mentioned	No	No	No	No	No
134	Neuroscience & Biobehavioral Reviews	ISSN (online): 1873-7528; Publisher: Elsevier	Not stated	Not stated /	Yes	Recommended/ advised/ suggested/ encouraged/ considered	Not mentioned	Yes: PRISMA (Preferred Reporting Items for Systematic Reviews and Meta-Analyses): Recommended/ advised/ suggested/ encouraged/ considered;	No	No	No	No
135	Neurosurgical Review	ISSN (online): 1437-2320; Publisher: Springer Nature	Not stated	COPE, ICMJE / Yes: Authorship	No		Not mentioned	Yes: PRISMA (Preferred Reporting Items for Systematic Reviews and Meta-Analyses): Recommended/ advised/ suggested/ encouraged/ considered; PRISMA-P: Recommended/ advised/ suggested/ encouraged/ considered	No	No	No	No
136	Nutrients	ISSN (online): 2072-6643; Publisher: MDPI	Not stated	COPE, ICMJE / Yes: Authorship, Methods	No	Not mentioned	Not mentioned	Yes: PRISMA (Preferred Reporting Items for Systematic Reviews and Meta-Analyses): Is required/ you must/ you must adhere to.../ should follow/ should be included/ expected to;	No	No	No	No
137	Nutrition Reviews	ISSN (online): 1753-4887; Publisher: Oxford Universtiy Press	Not stated	COPE, ICMJE, WAME / No, the role or commitment is not specified	Yes	Not mentioned	Not mentioned	Yes: PRISMA (Preferred Reporting Items for Systematic Reviews and Meta-Analyses): Is required/ you must/ you must adhere to.../ should follow/ should be included/ expected to; MOOSE (Meta-analyses of observational studies in epidemiology): Is required/ you must/ you must adhere to.../ should follow/ should be included/ expected to	Yes. The reporting checklist should be submitted upon submission	Yes: "Methods used to review and evaluate the literature using standardized procedures. This should include the databases used for the review, the key search terms, the criteria for excluding or including previous studies, and how the studies were evaluated and by whom."	No	Yes, Recommended/ advised/ suggested/ encouraged:"We suggest that data be presented in the main manuscript or additional supporting files, or deposited in a public repository whenever possible. This includes the complete list of all papers identified for systematic reviews whether they are used or not used for evaluating the literature. For information on general repositories for all data types, and a list of recommended repositories by subject area, please see Choosing where to archive your data."
138	Obesity Reviews	ISSN (online): 1467-789X; Publisher: Wiley	Not stated	COPE, ICMJE / Yes, Other: Conflicts of Interest	No	Not mentioned	Not mentioned	No	No	No	No	No
139	Obesity Surgery	ISSN (online): 1708-0428; Publisher: Springer Nature	Not stated	COPE, ICMJE / Yes: Authorship	No	Not mentioned	Not mentioned	No	No	No	No	No
140	Oral Diseases	ISSN (online): 1601-0825; Publisher: Wiley	Not stated	COPE, ICMJE / Yes: Authorship	No	Not mentioned	Not mentioned	Yes: PRISMA (Preferred Reporting Items for Systematic Reviews and Meta-Analyses): Is required/ you must/ you must adhere to.../ should follow/ should be included/ expected to;	Yes. The reporting checklist should be submitted upon submission	No	No	No

# of journals	Journal name	ISSN (online); Publisher	Publication date, or update date for author guideline (yyyymmdd or yyyymm if day is not available)	Publication ethics organisation(s) / Is there a particular aspect for which the ethics committee(s) is mentioned, and if so, is there a particular aspect mentioned?	Is there a separate headline/ section/ paragraph for systematic reviews in the author guideline?	Is registration of the protocol of the systematic review in PROSPERO or elsewhere mentioned?	Which methodology guidance is mentioned?	Is there a mention of a reporting standard for systematic reviews? If yes, which standards are mentioned?	Is a procedure to verify the reporting guidelines described?	Is reporting of the search methods for the systematic review mentioned specifically? (for example: number of databases, multiple reviewers, how to report your search strategy, …)	Is the involvement of an information specialist / medical librarian suggested in the author guidelines?	Is depositing / archiving / sharing of search strategies mentioned? (for example: depositing your search strategy on searchRxiv)
141	Palliative Medicine	ISSN (online): 1477-030X; Publisher: SAGE	Not stated	COPE, ICMJE / Yes: Authorship	Yes	Recommended/ advised/ suggested/ encouraged/ considered	Not mentioned	Yes: PRISMA (Preferred Reporting Items for Systematic Reviews and Meta-Analyses): Is required/ you must/ you must adhere to.../ should follow/ should be included/ expected to; Other: RAMESES or ENTREQ: Is required/ you must/ you must adhere to.../ should follow/ should be included/ expected to	Yes. The reporting checklist should be submitted upon submission	Yes: "All reviews should include sufficient detail on review question, inclusion and exclusion criteria, search strategies, data extraction and synthesis methods (as appropriate to the review design) for the study to be replicated. [...] Data sources: State the data sources used (including years searched)."	No	No
142	Patient Education & Counseling	ISSN (online): 0738-3991; Publisher: Elsevier	Not stated	Not stated /	No	Not mentioned	Not mentioned	Yes: PRISMA (Preferred Reporting Items for Systematic Reviews and Meta-Analyses): Recommended/ advised/ suggested/ encouraged/ considered;	No	No	No	No
143	Phytotherapy Research	ISSN (online): 1099-1573; Publisher: Wiley	Not stated	COPE / Yes, Other:Research and publication conduct	No	Not mentioned	Not mentioned	No	No	No	No	No
144	PLoS Medicine	ISSN (online): 1549-1676; Publisher: PLOS	Not stated	ICMJE / Yes: Authorship, Other: Sample references for Vancouver Style	Yes	Recommended/ advised/ suggested/ encouraged/ considered	Not mentioned	Yes: PRISMA (Preferred Reporting Items for Systematic Reviews and Meta-Analyses): Is required/ you must/ you must adhere to.../ should follow/ should be included/ expected to; PRISMA for Abstracts: Is required/ you must/ you must adhere to.../ should follow/ should be included/ expected to; EQUATOR Network: Is required/ you must/ you must adhere to.../ should follow/ should be included/ expected to	Yes. The reporting checklist should be submitted upon submission	No	No	No
145	PLoS Neglected Tropical Diseases	ISSN (online): 1935-2735; Publisher: PLOS	Not stated	ICMJE / Yes: Authorship, Other: Sample references for Vancouver Style	Yes	Recommended/ advised/ suggested/ encouraged/ considered	Not mentioned	Yes: PRISMA (Preferred Reporting Items for Systematic Reviews and Meta-Analyses): Is required/ you must/ you must adhere to.../ should follow/ should be included/ expected to; PRISMA for Abstracts: Is required/ you must/ you must adhere to.../ should follow/ should be included/ expected to; EQUATOR Network: Is required/ you must/ you must adhere to.../ should follow/ should be included/ expected to	Yes. The reporting checklist should be submitted upon submission	No	No	No
146	PLoS One	ISSN (online): 1932-6203; Publisher: PLOS	Not stated	ICMJE / Yes, Other:Sample references for Vancouver Style, patient privacy and informed consent	Yes	Recommended/ advised/ suggested/ encouraged/ considered	Not mentioned	Yes: PRISMA (Preferred Reporting Items for Systematic Reviews and Meta-Analyses): Is required/ you must/ you must adhere to.../ should follow/ should be included/ expected to; PRISMA-P: Is required/ you must/ you must adhere to.../ should follow/ should be included/ expected to; EQUATOR Network: Is required/ you must/ you must adhere to.../ should follow/ should be included/ expected to	Yes. The reporting checklist should be submitted upon submission	No	No	No
147	PLoS Pathogens	ISSN (online): 1553-7374; Publisher: PLOS	Not stated	ICMJE / Yes, Other: Sample references for Vancouver Style, patient privacy and informed consent	Yes	Not mentioned	Not mentioned	No	No	No	No	No
148	Postgraduate Medicine	ISSN (online): 1941-9260; Publisher: Taylor & Francis	20211202	ICMJE / Yes: Authorship, Data sharing, Other: Privacy and informed consent	No	Not mentioned	Not mentioned	No	No	No	No	No
149	Proceedings of the National Academy of Sciences of the United States of America (PNaS)	ISSN (online): 1091-6490; Publisher: National Academies Press (NAP)	202209	COPE / Yes, Other: Research Integrity	No	Not mentioned	Not mentioned	No	No	No	No	No
150	Progress in Neuro-Psychopharmacology & Biological Psychiatry	ISSN (online): 0278-5846; Publisher: Elsevier	Not stated	COPE, ICMJE, Other: Human research: The Code of Ethics of the World Medical Association (Declaration of Helsinki) / Yes: Methods	No	Not mentioned	Not mentioned	No	No	No	No	No
151	Psychiatry Research	ISSN (online): 0165-1781; Publisher: Elsevier	Not stated	COPE, ICMJE, Other: Human research: The Code of Ethics of the World Medical Association (Declaration of Helsinki) /	No	Not mentioned	Not mentioned	No	No	No	No	No

# of journals	Journal name	ISSN (online); Publisher	Publication date, or update date for author guideline (yyyymmdd or yyyymm if day is not available)	Publication ethics organisation(s) / Is there a particular aspect for which the ethics committee(s) is mentioned, and if so, is there a particular aspect mentioned?	Is there a separate headline/ section/ paragraph for systematic reviews in the author guideline?	Is registration of the protocol of the systematic review in PROSPERO or elsewhere mentioned?	Which methodology guidance is mentioned?	Is there a mention of a reporting standard for systematic reviews? If yes, which standards are mentioned?	Is a procedure to verify the reporting guidelines described?	Is reporting of the search methods for the systematic review mentioned specifically? (for example: number of databases, multiple reviewers, how to report your search strategy, …)	Is the involvement of an information specialist / medical librarian suggested in the author guidelines?	Is depositing / archiving / sharing of search strategies mentioned? (for example: depositing your search strategy on searchRxiv)
152	Resuscitation	ISSN (online): 0300-9572; Publisher: Elsevier	Not stated	ICMJE / No, the role or commitment is not specified	Yes	Not mentioned	Not mentioned	Yes: PRISMA (Preferred Reporting Items for Systematic Reviews and Meta-Analyses): Is required/ you must/ you must adhere to.../ should follow/ should be included/ expected to; EQUATOR Network: Is required/ you must/ you must adhere to.../ should follow/ should be included/ expected to	Yes. The reporting checklist should be submitted upon submission	No	No	No
153	Reviews in Medical Virology	ISSN (online): 1099-1654; Publisher: Wiley	Not stated	ICMJE / Yes, Other: Publication ethics	No	Not mentioned	Not mentioned	No	No	No	No	No
154	Scandinavian Journal of Clinical & Laboratory Investigation	ISSN (online): 1502-7686; Publisher: Taylor & Francis	20220816	Not stated /	No	Not mentioned	Not mentioned	No	No	No	No	No
155	Science of the Total Environment	ISSN (online): 0048-9697; Publisher: Elsevier	Not stated	COPE, ICMJE, Other: Human research: The Code of Ethics of the World Medical Association (Declaration of Helsinki) /	No	Not mentioned	Not mentioned	No	No	No	No	No
156	Scientific Reports	ISSN (online): 2045-2322; Publisher: Springer Nature	Not stated	Not stated /	No	Not mentioned	Not mentioned	No	No	No	No	No
157	Sensors	ISSN (online): 1424-8220; Publisher: MDPI	Not stated	COPE, ICMJE / Yes: Authorship, Methods, Other: Plagiarism	No	Not mentioned	Not mentioned	Yes: PRISMA (Preferred Reporting Items for Systematic Reviews and Meta-Analyses): Is required/ you must/ you must adhere to.../ should follow/ should be included/ expected to;	No	No	No	No
158	Signal Transduction and Targeted Therapy	ISSN (online): 2059-3635; Publisher: Springer Nature	20210726	COPE, ICMJE / Yes: Authorship, Other: Research Integrity	No	Not mentioned	Not mentioned	No	No	No	No	No
159	Sports Medicine	ISSN (online): 0112-1642; Publisher: Springer Nature	Not stated	COPE, ICMJE / Yes: Authorship	Yes	Not mentioned	Not mentioned	Yes: PRISMA (Preferred Reporting Items for Systematic Reviews and Meta-Analyses): Recommended/ advised/ suggested/ encouraged/ considered; PRISMA-P: Recommended/ advised/ suggested/ encouraged/ considered; EQUATOR Network: Recommended/ advised/ suggested/ encouraged/	No	No	No	No
160	Stroke	ISSN (online): 1524-4628; Publisher: Lippincott Williams & Wilkins	Not stated	Not stated /	No	Not mentioned	Not mentioned	Yes: PRISMA (Preferred Reporting Items for Systematic Reviews and Meta-Analyses): Is required/ you must/ you must adhere to.../ should follow/ should be included/ expected to; EQUATOR Network: Recommended/ advised/ suggested/ encouraged/ considered; MOOSE (Meta-analyses of observational studies in epidemiology): Is required/ you must/ you must adhere to.../ should follow/ should be included/ expected to	No	No	No	No
161	Supportive Care in Cancer	ISSN (online): 1433-7339; Publisher: Springer Nature	Not stated	COPE, ICMJE / Yes: Authorship	No	Not mentioned	Not mentioned	Yes: PRISMA (Preferred Reporting Items for Systematic Reviews and Meta-Analyses): Recommended/ advised/ suggested/ encouraged/ considered; PRISMA-P: Recommended/ advised/ suggested/ encouraged/ considered; EQUATOR Network: Recommended/ advised/ suggested/ encouraged/; MOOSE (Meta-analyses of observational studies in epidemiology): Recommended/ advised/ suggested/ encouraged/ considered	No	No	No	No
162	Surgery	ISSN (online): 0039-6060; Publisher: Elsevier	Not stated	COPE, ICMJE / Yes: Authorship, Other: References	No	Not mentioned	Not mentioned	Yes: PRISMA (Preferred Reporting Items for Systematic Reviews and Meta-Analyses): Is required/ you must/ you must adhere to.../ should follow/ should be included/ expected to;	No	No	No	No

# of journals	Journal name	ISSN (online); Publisher	Publication date, or update date for author guideline (yyyymmdd or yyyymm if day is not available)	Publication ethics organisation(s) / Is there a particular aspect for which the ethics committee(s) is mentioned, and if so, is there a particular aspect mentioned?	Is there a separate headline/ section/ paragraph for systematic reviews in the author guideline?	Is registration of the protocol of the systematic review in PROSPERO or elsewhere mentioned?	Which methodology guidance is mentioned?	Is there a mention of a reporting standard for systematic reviews? If yes, which standards are mentioned?	Is a procedure to verify the reporting guidelines described?	Is reporting of the search methods for the systematic review mentioned specifically? (for example: number of databases, multiple reviewers, how to report your search strategy, …)	Is the involvement of an information specialist / medical librarian suggested in the author guidelines?	Is depositing / archiving / sharing of search strategies mentioned? (for example: depositing your search strategy on searchRxiv)
163	Surgical Endoscopy	ISSN (online): 0930-2794; Publisher: Springer Nature	201806	ICMJE / Yes, Other: Conflicts of interest	No	Not mentioned	Not mentioned	No	No	No	No	No
164	Systematic Reviews	ISSN (online): 2046-4053; Publisher: Springer Nature	Not stated	COPE, ICMJE, WAME / No, the role or commitment is not specified	Yes	Recommended/ advised/ suggested/ encouraged/ considered	Not mentioned	Yes: PRISMA (Preferred Reporting Items for Systematic Reviews and Meta-Analyses): Is required/ you must/ you must adhere to.../ should follow/ should be included/ expected to; PRISMA-P: Is required/ you must/ you must adhere to.../ should follow/ should be included/ expected to; PRISMA for Abstracts: Is required/ you must/ you must adhere to.../ should follow/ should be included/ expected to	Yes. The reporting checklist should be submitted upon submission	No	No	Yes, Recommended/ advised/ suggested/ encouraged:"Authors are also encouraged to preserve search strings on searchRxiv https://searchrxiv.org/, an archive to support researchers to report, store and share their searches consistently and to enable them to review and re-use existing searches. searchRxiv enables researchers to obtain a digital object identifier (DOI) for their search, allowing it to be cited."
165	Trauma Violence & abuse	ISSN (online): 1552-8324; Publisher: SAGE	Not stated	Not stated /	No	Not mentioned	Not mentioned	No	No	Yes: "Each manuscript must: include […] criteria for inclusion, how research studies were identified […]"	No	No
166	Travel Medicine and infectious Disease	ISSN (online): 1873-0442; Publisher: Elsevier	Not stated	COPE, ICMJE, Other: Human research: The Code of Ethics of the World Medical Association (Declaration of Helsinki) /	Yes	Recommended/ advised/ suggested/ encouraged/ considered	Cochrane Handbook for Systematic Reviews of Interventions: recommended/ advised/ suggested/ encouraged/ considered	Yes: PRISMA (Preferred Reporting Items for Systematic Reviews and Meta-Analyses): Recommended/ advised/ suggested/ encouraged/ considered; EQUATOR Network: Recommended/ advised/ suggested/ encouraged/ considered	No	No	No	No
167	Vaccine	ISSN (online): 1873-2518; Publisher: Elsevier	Not stated	COPE, ICMJE, Other: Human research: The Code of Ethics of the World Medical Association (Declaration of Helsinki) / Yes: Authorship	No	Not mentioned	Not mentioned	No	No	No	No	No
168	World Neurosurgery	ISSN (online): 1878-8769; Publisher: Elsevier	Not stated	COPE, ICMJE, Other: Human research: The Code of Ethics of the World Medical Association (Declaration of Helsinki) /	No	Not mentioned	Not mentioned	No	No	No	No	No
